# Cephalic arch restenosis rates in hemodialysis patients with brachiocephalic fistulae: a retrospective multicenter analysis of 3301 patients

**DOI:** 10.1186/s12882-022-02728-4

**Published:** 2022-03-18

**Authors:** Rishi N. Razdan, Melvin Rosenblatt, Yue Jiao, Nancy McLaughlin, Len A. Usvyat, Murat Sor, John W. Larkin

**Affiliations:** 1 Azura Vascular Care, Malvern, PA USA; 2 Fresenius Medical Care, Global Medical Office, Waltham, MA USA; 3 Georgetown University, Washington, DC USA

**Keywords:** Cephalic arch, Stenosis, Brachiocephalic fistula, Arteriovenous fistula, End stage renal disease, Angioplasty, Stent

## Abstract

**Background:**

We evaluated restenosis rates at the cephalic arch after percutaneous angioplasty and stenting procedures in patients with brachial artery to cephalic vein arteriovenous fistula (BCAVF) hemodialysis access.

**Methods:**

We used data from adult hemodialysis patients treated at a national network of 44 outpatient interventional facilities during Oct 2011–2015. We included data from patients with BCAVF who received an exclusive angioplasty, or stent with angioplasty, for treatment of cephalic arch stenosis and had ≥1 subsequent evaluation of the cephalic arch. Median percent restenosis per month at cephalic arch and days between encounters was calculated from the 1st index to 2nd procedure, and for up to 4 subsequent encounters. Analyses were stratified by intervention and device types.

**Results:**

We identified a cohort of 3301 patients (mean age 62.2 ± 13.9 years, 58.5% male, 33.2% white race) with a BCAVF who had an angioplasty, or stent, at the cephalic arch for an index and ≥ 1 follow-up procedure. Between the 1st index to 2nd procedure, patients who received an angioplasty (*n* = 2663) or stent (*n* = 933) showed a median decrease of 18.9 and 16.5% in luminal diameter per month and a median time of 93 and 91 days between encounters, respectively. Restenosis and day rates were similar for standard versus high-pressure angioplasties. Bare metal stents showed 10.1 percentage point higher restenosis rate compared to stent grafts. Restenosis rates and time to restenosis were relatively consistent across subsequent encounters.

**Conclusions:**

Findings suggest hemodialysis patients with a BCAVF who require an angioplasty or stent to treat a stenosis at the cephalic arch will have stenosis reformed at a rate of 18.9 and 16.5% per month after the first intervention, respectively. Findings suggest patients are at risk of having significant lesions at the cephalic arch within 3 months after the previous intervention.

**Supplementary Information:**

The online version contains supplementary material available at 10.1186/s12882-022-02728-4.

## Background

In the United States, more than 785,000 people are diagnosed with kidney failure and require kidney replacement therapy (KRT) to sustain life [1]. About 62% of patients are treated by the KRT of hemodialysis, whereby blood is filtered three or more times per week using a vascular access. Dialysis access types include arteriovenous fistulae (AVFs), arteriovenous grafts (AVGs), or catheters. Of these options, AVFs are considered the preferred access given better function and lower infection and mortality rates compared to catheters and AVGs [2]. Also, studies have shown that AVFs demonstrate lower thrombosis and access failure rates than AVGs [3]. Among prevalent hemodialysis patients, about 66% use an AVF [1].

The three most common AVFs are the radio-cephalic fistula, brachial artery to cephalic vein fistula, and brachial artery to basilic vein transposed fistula. Although the first choice is the radio-cephalic forearm fistula, the brachial artery to cephalic vein fistula, or brachiocephalic fistula (BCAVF), is one of the most popular types for many reasons. Among several advantages is ease of creation, high maturation rates, and high flow rates. Disadvantages include higher rates of steal syndrome and symptomatic central venous stenosis [4].

With an increasing prevalence of BCAVF comes unique complications requiring treatment to avoid failure. For BCAVF, the typical site of stenosis is the cephalic arch [4–7]. Potential etiologies for stenosis and subsequent restenosis at the cephalic arch include increased flow in an outflow vein, external compression by fascia and pectoralis major, angulation, numerous valves in the outflow vein, and biochemical changes associated with kidney failure. The cephalic arch is typically a single channel joining with the axillary vein, yet variant anatomy such as a bifid arch, or abnormal termination point (internal or external jugular veins), can cause venous outflow obstruction and access malfunction [8]. It is likely a combination of multiple factors contribute to occurrence and recurrence of stenosis at this site.

To date, there are no large multicenter studies describing the treatment and effectiveness of percutaneous interventions at the cephalic arch in BCAVFs. Our study aimed to evaluate cephalic arch restenosis rates after percutaneous intervention, including transluminal balloon angioplasty and stenting.

## Methods

### Setting

We used data from a network of 44 outpatient vascular care and ambulatory surgery centers (Azura Vascular Care, Malvern, PA) across the United States between October-2011 through October-2015. This analysis was conducted under a protocol approved by New England Independent Review Board (IRB). The IRB determined this analysis of deidentified patient data was exempt and did not require informed consent per the United States Code of Federal Regulations 45CFR46 (Needham Heights, MA; NEIRB#WO-1-574-1). This study adhered with the Declaration of Helsinki.

### Patient population

We included data from adult patients (age ≥ 18 years) with a brachiocephalic AVF (BCAVF) who: 1) were referred for evaluation of an access malfunction, 2) received an angioplasty or stent for treatment of stenosis at the cephalic arch, and 3) had ≥1 subsequent evaluation of the cephalic arch. After the initial referral, subsequent encounters were either a clinically timed evaluation scheduled by the interventionalist, or another referral for an access malfunction from the dialysis center (e.g. prolonged bleeding, high venous pressures, low access flows). We excluded data from patients who received a coinciding thrombectomy, pharmacomechanical thrombolysis, or embolization at the same encounter as an angioplasty/stent. The dataset also did not contain patients who had an access ligation or excision after initial referral visit.

### Standard of care practices

Patient care was performed under the provider’s standard operating procedures by interventional radiologists, interventional nephrologists, and vascular surgeons who specialize in dialysis access care. A universal electronic medical record system was used to record encounters/procedures. Patients referred for evaluation of a malfunctioning AVF had historical and physical examination performed prior to intervention. During patient visits, the need for an angiogram was determined in a manner consistent with national guidelines, [9] and based on abnormalities identified during the physical examination, as well as complaints/subjective reports from the patient/dialysis center (e.g. increased pulsatility during physical exam combined with prolonged bleeding after hemodialysis). When angiogram was appropriate, intravenous fentanyl and midazolam were used for moderate sedation under nurse/anesthesiologist monitoring; propofol was also administered in rare instances. A combination of fluoroscopy with contrast and ultrasound was used for evaluation of fistulae. Percutaneous intervention(s) were performed on clinically significant lesions found to have stenosis with > 50% narrowing in luminal diameter as compared by the diameter of the nearest normal appearing vein found during two dimensional angiogram; these practices were consistent with national guidelines (i.e. Kidney Disease Outcomes Quality Initiative Clinical Practice Guideline for Vascular Access) [9]. The provider’s clinics did not use three-dimensional computed tomography angiography in standard practices, and therefore did not measure luminal area. The need for and timing of clinically timed evaluations after any given angioplasty or stent procedure was based on the medical judgment of the interventionalist.

Standard practice was typically to treat detected lesions with standard angioplasty (< 24 atm (ATM) rated burst pressure balloon) until stenosis was eliminated using inflation times based on operator discretion. When necessary, high-pressure angioplasty (> 24 ATM rated burst pressure balloon) were recommended to eliminate a lesion if resistant to standard angioplasty, or a known history of a lesion at the site requiring high-pressure angioplasty. Balloon size ranged from 4-to-14 mm in diameter. If a residual stenosis was identified, or if a stenosis was unresponsive to angioplasty, a stent or stent graft was placed. Stent size was recommended to be based upon comparison of the normal appearing vein with oversizing by 20–40% to accommodate for vein elasticity and position the stent. A post-stent deployment angioplasty could be performed to further expand the stent as clinically indicated.

### Cephalic arch stenosis

Cephalic arch was defined as the portion of the cephalic vein within 5 cm of the confluence with the axillary vein, or larger outflow vein in instances of aberrant anatomy. Stenoses were assessed comparing the narrowest luminal diameter at the cephalic arch, as a percentage of the nearest normal appearing vein. Percent stenosis was documented before and after each intervention.

### Analysis design

#### Analysis of restenosis rates by intervention type

We assessed data from patients who received either exclusively an angioplasty, or a stent with an angioplasty, for treatment of stenosis at the cephalic arch at each index procedure. The first encounter defined the 1st index procedure for patients who had an angioplasty without any stent placement. The first stent placement defined the 1st index procedure for patients who received a stent with an angioplasty.

For each intervention type (angioplasty or stent group), we calculated the per patient difference in the percent stenosis after the 1st index procedure (post-intervention) to the percent stenosis before the 2nd procedure (pre-intervention). We computed restenosis rates via the change in the percent stenosis per month from the 1st index to 2nd procedure. The equation below shows a mathematical description of the restenosis rate described above.

$$Restenosis\ rate\ per\ month=\frac{Xi- Xf}{\varDelta t/30.5}$$

Where *Xi* = Percent stenosis at the follow up visit before any intervention was performed, *Xf* = Percent stenosis at the 1st index visit after the angioplasty or stent intervention was completed, and *Δt* = equals the days from the 1st index procedure to the 2nd procedure. The calculation of the restenosis rate defined 30.5 days per month.

Consistent calculations were made between subsequent encounters (2nd index to 3rd, 3rd index to 4th procedure). Rates were reported as median value and interquartile range (lower (25%) and upper (75%) percentile) and distribution data was plotted in categories of restenosis (0%, > 0 to ≤5%, > 5 to ≤10%, > 10 to ≤15%, > 15 to ≤20%, > 20 to ≤25%, > 25 to ≤30%, > 30 to ≤35%, > 35 to ≤40%, > 40 to ≤45%, > 45 to ≤50%, > 50 to ≤100%, or > 100% stenosis per month).

Within the angioplasty group defined at the 1st index procedure, restenosis rates were calculated for the sub-group who had an exclusive angioplasty at the 2nd index to 3rd, as well as the 3rd index to 4th, procedures. Among the stent group, restenosis rates were calculated for the sub-groups who received an angioplasty without any stent at the subsequent 2nd index to 3rd and 3rd index to 4th procedures, or another stent at the subsequent 2nd index to 3rd and 3rd index to 4th procedures.

#### Analysis of restenosis rates by intervention and device types

We stratified the analysis for each intervention type by the device type used across visits. For angioplasty group, device types were classified as standard or high-pressure. For stent group, device types were classified as bare metal and stent graft (covered stent).

We further assessed the subset who received an index stent at the 1st visit, and a subsequent exclusive angioplasty at the 2nd index and/or 3rd index visits. For this, we evaluated restenosis rates overall and by device type of the stent at the 1st index visit, as well as the device type of the angioplasty at the 2nd index and 3rd index visits.

## Results

### Patient characteristics

**Fig. 1:**
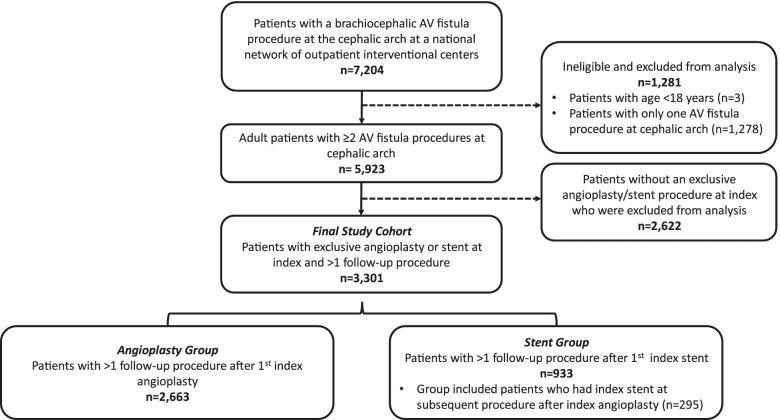
Patient Flow Diagram

We identified 3301 eligible patients with a brachiocephalic AVF (BCAVF) who had an exclusive angioplasty, or a stent with an angioplasty, at the cephalic arch for an index procedure and ≥ 1 follow-up procedure(s) during the 4-years (Fig. 1). Cohort was on average 62.2 ± 13.9 years old, 58.5% male, 33.2% white race, 30.9% black race, and 75.9% had a left AVF (Table 1). On average patients had 5.0 ± 3.2 procedures at the cephalic arch across follow-up.

**Table 1:** Patient Characteristics

Parameter	N (%), Mean ± SD
Eligible Adult Patients	3301 (100)
Age years	62.2 ± 13.9
Male	1930 (58.5)
Race	
*White*	1097 (33.2)
*Black*	1019 (30.9)
*Asian*	53 (1.6)
*Other*	46 (1.4)
*Unknown*	1086 (32.9)
Ethnicity	
*Not Hispanic*	1731 (52.4)
*Hispanic*	484 (14.7)
*Unknown*	1086 (32.9)
AV Fistula Lateral Location	
*Left*	2504 (75.9)
*Right*	785 (23.8)
*Unknown*	12 (0.4)
Total Number of Procedures in Cohort	16,615
Procedures per Patient	5.03 ± 3.15

*N* patient number, *SD* standard deviation

### Restenosis rates after angioplasty

**Fig. 2:**
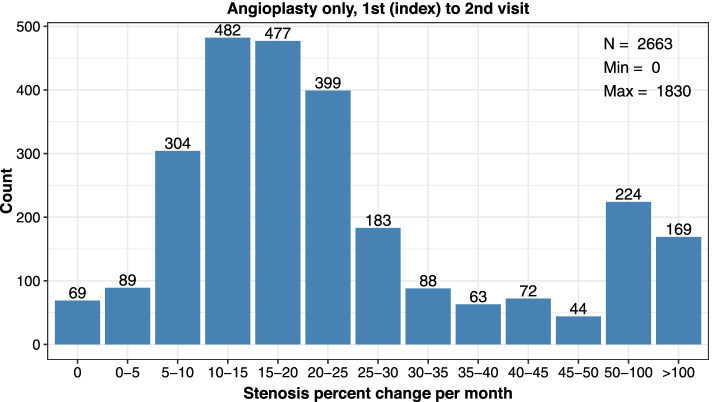
Distribution of restenosis rates (% decrease in lumen diameter per month) at the cephalic arch after the 1st index to 2nd visits among patients treated with angioplasty

There were 2663 patients who received an exclusive angioplasty without a stent for treatment of stenosis at the cephalic arch during the 1st index visit and returned for a 2nd follow-up visit. Between the 1st index to 2nd visit, this group showed a median decrease of 18.9% in luminal diameter per month and a median time of 93 days between encounters (Table 2). The distributions of restenosis rates per month after an angioplasty are shown in Fig. 2 and revealed 14.8% of patients had a recurrent lesion with > 50% stenosis per month (i.e. [393 patients with stenosis % per month > 50% / 2663 total patients] * 100 = 14.8%), which represents the subset of patients with a clinically meaningful dysfunction in patency within a month.

**Table 2:** Cephalic Arch Restenosis Rates Over Time by Intervention Type

**Angioplasty without Stent**	**Visit Pair**		
	**1st to 2nd**	**2nd to 3rd**	**3rd to 4th**
*Parameter*	*n* = 2663	*n* = 1513	*n* = 901
*Median % Decrease in Lumen Diameter per Month (IQR)*	18.9 (12.3–30.0)	18.4 (12.7–25.1)	17.8 (12.9–24.2)
*Median Days between Encounters (IQR)*	93 (58–128)	96 (75–124)	94 (84–119)
**Stent**	**Visit Pair**		
	**1st to 2nd**	**2nd to 3rd**	**3rd to 4th**
*Parameter*	*n* = 933	*n* = 48	*n* = 5
*Median % Decrease in Lumen Diameter per Month (IQR)*	16.5 (7.5–27.2)	19.1 (14.6–29.1)	19.8 (13.0–24.6)
*Median Days between Encounters (IQR)*	91 (42–126)	91 (59–115)	95 (93–100)

*IQR* Interquartile range showing the 25 to 75% percentile

A subset of 56.8 and 33.8% of patients received a subsequent exclusive angioplasty to treat restenosis at the 2nd index visit and 3rd index visit, respectively. Between the 2nd index to 3rd and 3rd index to 4th visits, patients showed a median decrease of 18.4 and 17.8% in luminal diameter per month and a median time of 96 and 94 days between encounters (Table 2). The distribution of restenosis rates showed 9.6% of patients between the 2nd index to 3rd visits (Additional file 1) and 7.4% of patients between the 3rd index to 4th visits (Additional file 2) exhibited > 50% stenosis per month, showing decreasing trends in the proportion of patients with a clinically meaningful dysfunction in patency each month with additional angioplasties. There were 11.1% of patients with an exclusive angioplasty at the 1st index visit who received a stent at the subsequent 2nd or 3rd index visit. These patients were included in the stent group as of the index encounter where they received a stent.

**Fig. 3:**
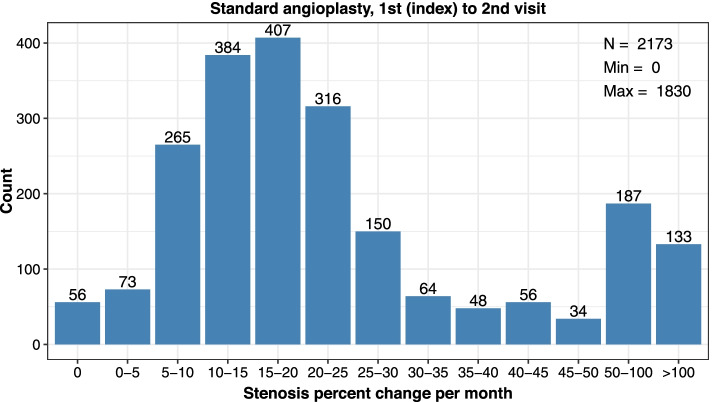
Distribution of restenosis rates (% decrease in lumen diameter per month) at the cephalic arch after the 1st index to 2nd visits among patients treated with standard angioplasty

**Fig. 4:**
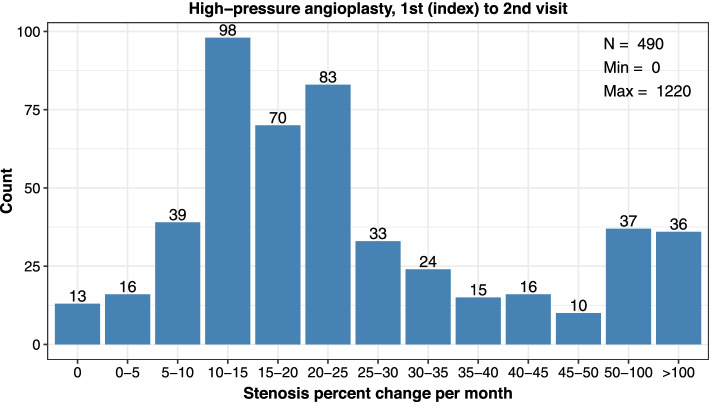
Distribution of restenosis rates (% decrease in lumen diameter per month) at the cephalic arch after the 1st index to 2nd visits among patients treated with high-pressure angioplasty

Assessment of angioplasty types showed standard balloons were generally utilized. During the 1st index visit, 18.4% of angioplasties used a high-pressure balloon and the remainder used standard balloons; high-pressure balloons were used in 22.5 and 23.8% of angioplasties at the 2nd index and 3rd index visits (Table 3). Restenosis rates were similar between angioplasty types and median days between encounters were the same at the 1st index to 2nd visit (Table 3; Figs. 3 and 4), yet progressively increased to differ by 5 days more in the standard versus high-pressure angioplasty groups between the 3rd to 4th visit (Table 3; Additional files 3, 4, 5 and 6).

**Table 3:** Cephalic Arch Restenosis Rates Over Time by Angioplasty Type

**Standard Angioplasty**	**Visit Pair**		
	**1st to 2nd**	**2nd to 3rd**	**3rd to 4th**
*Parameter*	*n* = 2173	*n* = 1172	*n* = 687
*Median % Decrease in Lumen Diameter per Month (IQR)*	18.6 (12.2–29.0)	18.4 (12.7–25.1)	17.1 (12.4–23.3)
*Median Days between Encounters (IQR)*	93 (60–126)	95 (77–121)	96 (85–121)
**High-Pressure Angioplasty**	**Visit Pair**		
	**1st to 2nd**	**2nd to 3rd**	**3rd to 4th**
*Parameter*	*n* = 490	*n* = 341	*n* = 214
*Median % Decrease in Lumen Diameter per Month (IQR)*	20.3 (13.1–32.8)	19.3 (12.8–28.5)	19.2 (14.2–28.2)
*Median Days between Encounters (IQR)*	93 (56–133)	98 (68–133)	91 (72–114)

*IQR* Interquartile range showing the 25 to 75% percentile

### Restenosis rates after stenting

**Fig. 5:**
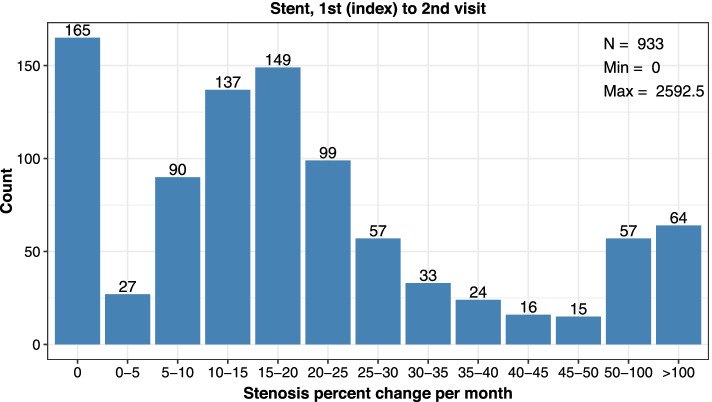
Distribution of restenosis rates (% decrease in lumen diameter per month) at the cephalic arch after the 1st index to 2nd visits among patients treated with stent

There were 933 patients who received a stent for treatment of stenosis at the cephalic arch with a subsequent follow-up encounter. During the 1st index stent to 2nd visit, these patients exhibited a median decrease of 16.5% per month in luminal diameter and had a median of 91 days between subsequent encounters (Table 2). The distribution data showed 13.0% of patients had recurrence of > 50% stenosis per month after receiving a stent (Fig. 5).

Within this group of patients who received a 1st index stent, a subset of 5.1 and 0.5% of patients received a stent at the 2nd index visit and 3rd index visit, respectively. These patients had a median decrease of 19.1 and 19.8% in luminal diameter per month and a median time of 91 and 95 days between encounters from the 2nd index to 3rd and 3rd index to 4th visits (Table 2), and the distribution data showed trends for decreasing proportion with > 50% stenosis (Additional files 7 and 8).

**Fig. 6:**
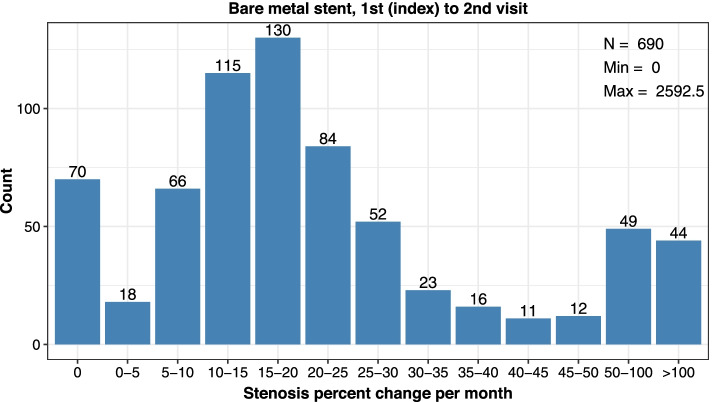
Distribution of restenosis rates (% decrease in lumen diameter per month) at the cephalic arch after the 1st index to 2nd visits among patients treated with bare metal stent

**Fig. 7:**
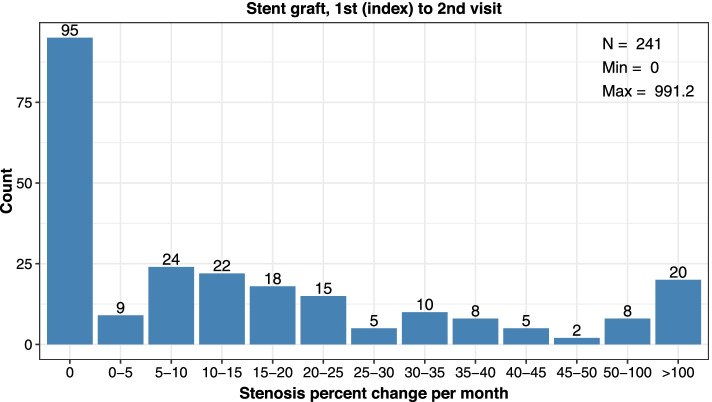
Distribution of restenosis rates (% decrease in lumen diameter per month) at the cephalic arch after the 1st index to 2nd visits among patients treated with stent graft

Assessment of stent types showed bare metal stents were more commonly used. At the 1st index visit, 25.8% of stenting procedures used a stent graft and 74.0% used a bare metal stent (Table 4). Two patients did not have documentation of stent type and were not included. Stent grafts were used in 18.8% of stenting procedures at the 2nd index visit and were never utilized at the 3rd index procedure. Between the 1st index to 2nd visit, patients who received a bare metal stent showed a remarkably higher restenosis rate per month (10.1 percentage points higher) yet showed more days between encounters (5 more days) as compared to patients who received a stent graft (Table 4; Figs. 6 and 7). This difference in restenosis rates became smaller by stent types in the 2nd index to 3rd visit, where patients who received a stent graft had 5.4 percentage point lower restenosis rate per month and the same days between encounters, as compared to bare metal stents (Table 4; Additional files 9, 10 and 11). Notably, there was only a select group of patients (*n* = 9) who received a stent graft at a 2nd procedure.

**Table 4:** Cephalic Arch Restenosis Rates Over Time by Stent Type

**Bare Metal Stent**	**Visit Pair**		
	**1st to 2nd**	**2nd to 3rd**	**3rd to 4th**
*Parameter*	*n* = 690	*n* = 39	*n* = 5
*Median % Decrease in Lumen Diameter per Month (IQR)*	18.1 (10.9–27.6)	20.0 (15.7–29.7)	19.8 (13.0–24.6)
*Median Days between Encounters (IQR)*	91 (54–125)	91 (60–107)	95 (93–100)
**Stent Graft**	**Visit Pair**		
	**1st to 2nd**	**2nd to 3rd**	**3rd to 4th**
*Parameter*	*n* = 241	*n* = 9	*n* = 0
*Median % Decrease in Lumen Diameter per Month (IQR)*	8.0 (0.0–23.6)	14.6 (0.0–18.8)	
*Median Days between Encounters (IQR)*	86 (29–130)	91 (58–133)	

*IQR* Interquartile range showing the 25 to 75% percentile

### Restenosis rates among patients with an index stent and subsequent angioplasty

Among the stent group defined at the 1st index visit, 43.9 and 20.5% received an exclusive angioplasty without a stent at the subsequent 2nd index and 3rd index visits, respectively (Table 5). Assessment of restenosis rates in the subsets of patients between the 2nd index to 3rd and 3rd index to 4th visits showed a decrease of 18.7 and 18.4% in the luminal diameter per month and a median time of 91 and 93 days between encounters (Table 5; Additional files 12 and 13).

**Table 5:** Cephalic Arch Restenosis Rates Over Time among Patients with an Index Stent and Subsequent Angioplasty

**Any Stent Type at 1st Index Visit, and Any Angioplasty Type at 2nd Index or 3rd Index Visits**	**Visit Pair**	
	**2nd to 3rd**	**3rd to 4th**
*Parameter*	*n* = 410	*n* = 191
*Median % Decrease in Lumen Diameter per Month (IQR)*	18.7 (11.9–25.4)	18.4 (12.6–24.2)
*Median Days between Encounters (IQR)*	91 (68–119)	93 (70–121)
**Any Stent Type at 1st Index Visit, and Standard Angioplasty at 2nd Index or 3rd Index Visits**	**Visit Pair**	
	**2nd to 3rd**	**3rd to 4th**
*Parameter*	*n* = 337	*n* = 155
*Median % Decrease in Lumen Diameter per Month (IQR)*	18.0 (11.6–25.1)	17.4 (12.3–23.3)
*Median Days between Encounters (IQR)*	93 (77–121)	98 (78–121)
**Any Stent Type at 1st Index Visit, and High-Pressure Angioplasty at 2nd Index or 3rd Index Visits**	**Visit Pair**	
	**2nd to 3rd**	**3rd to 4th**
*Parameter*	*n* = 73	*n* = 36
*Median % Decrease in Lumen Diameter per Month (IQR)*	20.9 (15.4–26.9)	19.7 (14.4–29.4)
*Median Days between Encounters (IQR)*	91 (63–100)	91 (62–106)
**Bare Metal Stent at 1st Index Visit, and Any Angioplasty Type at 2nd Index or 3rd Index Visits**	**Visit Pair**	
	**2nd to 3rd**	**3rd to 4th**
*Parameter*	*n* = 332	*n* = 149
*Median % Decrease in Lumen Diameter per Month (IQR)*	19.1 (12.5–25.4)	18.4 (13.3–24.2)
*Median Days between Encounters (IQR)*	91 (70–119)	93 (70–119)
**Stent Graft at 1st Index Visit, and Any Angioplasty Type at 2nd Index or 3rd Index Visits**	**Visit Pair**	
	**2nd to 3rd**	**3rd to 4th**
*Parameter*	*n* = 76	*n* = 42
*Median % Decrease in Lumen Diameter per Month (IQR)*	16.1 (10.7–25.4)	17.6 (11.3–23.5)
*Median Days between Encounters (IQR)*	93 (68–133)	91 (72–126)

*IQR* Interquartile range showing the 25 to 75% percentile

Assessment by the angioplasty type used at the subsequent visits after an index stent procedure showed 17.8 and 18.8% of angioplasties used a high-pressure balloon at the 2nd index and 3rd index visits, respectively, and the remainder used a standard balloon. Restenosis rates were similar among patients who received a high-pressure angioplasty versus standard angioplasty and were consistent with restenosis rates among patients who never had a prior stenting procedure (Table 5; Additional files 14, 15, 16 and 17). Among patients who received a high-pressure angioplasty, median time between encounters was 2 and 7 days shorter between the 2nd index to 3rd and 3rd index to 4th visits, respectively, as compared to those who received a standard angioplasty.

Further assessment of restenosis rates associated with subsequent angioplasty procedures after an index stenting were performed by stent type. Subsequent angioplasties after a stent graft at the 1st index visit were found to have a slightly lower restenosis rate per month (3 percentage points lower) between the 2nd index to 3rd visits with consistent days between encounters, as compared to bare metal stent. Restenosis rates and days were relatively similar between the 3rd index to 4th visits (Table 5; Additional files 18, 19, 20 and 21).

## Discussion

One of the most common, and easily addressed, complications with AVFs is development of primary and recurrent stenoses in various sites of the vascular access [10]. Each AVF type behaves differently as to where stenoses typically occur. Brachiocephalic AVFs (BCAVFs) are typically plagued by stenoses at the cephalic arch, with reports describing up to a 77% prevalence of significant stenoses at this site [11]. Angioplasty has been a mainstay of treatment for dialysis access stenoses, although literature has supported the use of stent graft placement compared with angioplasty alone [12–15].

We found the luminal diameter at the cephalic arch decreased by 18.9% per month after the first exclusive angioplasty and 16.5% per month after the first stenting procedure among patients with a BCAVF. Restenosis rates were relatively similar between high-pressure and standard angioplasty procedures, suggesting consistent effectiveness when a high-pressure balloon was required to eliminate the lesion. Restenosis rates had trends to be higher after the first bare metal stent (18.1% per month) compared to stent graft (8.0% per month). These signals are consistent with a meta-analysis including 457 patients with a BCAVF who had an intervention at the cephalic arch; this analysis showed significantly higher patency at 6 and 12 months for stent grafts versus bare metal stents and angioplasty alone [14]. Overall, our findings indicate that once a patient presents with a cephalic arch stenosis and is treated, they are at risk of having a 50% stenosis in 2.65 months after an angioplasty (50% stenosis / 18.9% restenosis per month = 2.65 months) or 3.03 months after a stent (50% stenosis / 16.5% restenosis per month = 3.03 months). Stated differently, a patient with a post intervention residual stenosis of 10% after their first exclusive angioplasty or stent is estimated to progress to have a 67% or 60% stenosis in 3 months, respectively. Restenosis rates were relatively similar across subsequent angioplasty or stent procedures during follow-up, yet the proportion with a stenosis > 50% per month had trends to be lower with more interventions. This indicates there were typically fewer extremely high restenosis rates with more interventions, as well as less variation in the rate.

One of the longest retrospective studies involving hemodialysis upper arm AVFs found a 34% primary and 82% secondary patency rate at one year, although there was no differentiation between brachiocephalic versus brachiobasilic fistulae [6]. This study found the interval for re-intervention was 10.6 months in upper arm AVFs, but did not specify the exclusivity or combinations of intervention types nor the site of malfunction. We observed the interval for re-intervention after exclusive angioplasty or stenting procedures was approximately 3 months. However, the interquartile range for restenosis rates and re-intervention times were typically large, which represents high patient-to-patient variability.

The predictors of restenosis risk at the cephalic arch have not been established and future investigations are needed, optimally with more robust data from all types of interventional locations as well as the dialysis clinics, and include data on patient characteristics, interventions, and hemodialysis treatments. Arterialized pressures, anatomical orientation, intimal hyperplasia, and turbulence are likely culprits for rapidly recurrent and possibly angioplasty-resistant stenoses [16]. Fistula age may also be affecting the durability of the vessel wall [6]. Neo-intimal hyperplasia and turbulence has been suggested as a cause of rapid recurrent in-stent stenosis [12, 17, 18]. For bare metal stents, the intima continues to be exposed to turbulent flow and biochemical constituents of blood vessels through the interstices [18]. This may explain why lower restenosis rates were seen for stent grafts that cover the intima with a physical barrier while propping open the vessel lumen, nonetheless, the intima at the ends of stent grafts remains exposed and hyperplasia can be a point of recurrent stenosis due to the same reasons [19, 20]. Although not evaluated, several incidences of stent fracture were noted as causing recurrent stenosis; oftentimes, angioplasty and/or stenting was required to improve the flow and appearance.

Recurrent stenosis and repeated interventions are common at the cephalic arch in BCAVFs, and alternative treatments may be worthwhile to be considered in appropriate cases where angioplasty has been ineffective. These include stents, with stent grafts appearing to have the most favorable outcomes in our analysis. Stent grafts have also been previously shown to be more effective in the treatment of lesions in AVFs and AVGs, as compared to bare metal stents and angioplasty alone [12–15, 21–23]. Stent grafts can be constructed from various materials (e.g. metals, plastics, woven polyester), but the differences between restenosis rates with stent graft types is speculative. Other alternative treatments include percutaneous drug eluting balloons, as well as other surgical procedures including cephalic arch turn-down and cut-down and patch angioplasty. These procedures were not evaluated in this study due to minimal-to-no use in the outpatient setting. With respect to drug eluting balloons, their use at the time of the study was not widespread secondary to cost of equipment and relatively little data on their outcomes during the time of the study.

Our study has several strengths, the most obvious of which includes the focus on exclusive interventions that afforded the ability to reasonably assess restenosis rates at a specific lesion site. In addition, the analysis included a large number of patients treated by various practitioner types in a broad geography. All these factors represent clinically relevant interventions and consequently, this data likely represents reproducible, real-world outcomes. However, this study cohort may not be fully generalizable and is not representative of the groups of patients who had unremarkable complications upon evaluation, or a mixture of interventions (e.g. thrombectomy and high-pressure angioplasty). We cannot rule out a potential bias by indication for angiogram. We did not have information on patients who did not require a subsequent intervention after their first procedure, which included more than 1200 patients who received only one measurement at the cephalic arch during a mixture of evaluations and/or intervention types. Furthermore, the provider’s centers did not routinely perform ultrasounds before inventions to provide further details. Nonetheless, the cohort included a reasonable number of patients with minimal to no restenosis after an index angioplasty or stent likely making the results generalizable to what occurs in treatment of access stenoses in BCAVFs. These results are not generalizable to all access failures, given the high potential for multifactorial causes including formation of a thrombus and blood vessels diverging into abnormal vascular channels. Further independent studies are needed to define the rates and risk factors of recurrent complications for those events linked to thrombosis of the access.

The retrospective nature of this study is an inherent weakness. We did not have access to data from the dialysis centers and several factors such as the time the BCAVF was in use may have the potential influence the results. Also, stenosis measurements may have varied by practitioner, although a small random sampling of cases with image review showed good agreement with reported degrees of stenosis. Given the large number of centers, inter-operator variability also poses a problem when evaluating the need for stenting, stent type, and general personal practice regarding stenting versus angioplasty alone. Also, the precise morphology of the stenosis, focal versus long segment versus multifocal, was not clearly specified. While morphology may play a role in the long-term success of endoluminal interventions, it is not clear stratifying the results based on morphology would have any impact. Furthermore, these models of recurrence are based on a linear progression of stenosis development warranting treatment. It is feasible that after numerous interventions, the rate of progression may change, either for better or worse.

## Conclusions

In conclusion, the study demonstrated restenosis rates at the cephalic arch in hemodialysis patients with a brachiocephalic fistula requiring an angioplasty or stent were 18.9 and 16.5% per month after the first intervention, respectively. Restenosis rates appeared to be relatively consistent across subsequent procedures. Findings suggest patients are at risk of having significant lesions (> 50% stenosis) within 3 months after the previous intervention at the cephalic arch. When clinically indicated, use of stent grafts seemed to lessen recurrent stenosis, especially if used as the first stent. It appears prudent to have patients who develop a cephalic arch lesion return for an evaluation after approximately 3 months. At that time, a careful history and physical exam should be performed to determine if recurrence is present. If the assessment indicates such, imaging with possible intervention is recommended to maximize patency and functionality of a patient’s hemodialysis access, thereby prolonging life.

## Supplementary Information

**Additional file 1.** Supplemental figure on distribution of restenosis rates (% decrease in lumen diameter per month) at the cephalic arch after the 2nd index to 3rd visits among patients treated with angioplasty

**Additional file 2.** Supplemental figure on distribution of restenosis rates (% decrease in lumen diameter per month) at the cephalic arch after the 3rd index to 4th visits among patients treated with angioplasty

**Additional file 3.** Supplemental figure on distribution of restenosis rates (% decrease in lumen diameter per month) at the cephalic arch after the 2nd index to 3rd visits among patients treated with standard angioplasty

**Additional file 4.** Supplemental figure on distribution of restenosis rates (% decrease in lumen diameter per month) at the cephalic arch after the 3rd index to 4th visits among patients treated with standard angioplasty

**Additional file 5.** Supplemental figure on distribution of restenosis rates (% decrease in lumen diameter per month) at the cephalic arch after the 2nd index to 3rd visits among patients treated with high-pressure angioplasty

**Additional file 6.** Supplemental figure on distribution of restenosis rates (% decrease in lumen diameter per month) at the cephalic arch after the 3rd index to 4th visits among patients treated with high-pressure angioplasty

**Additional file 7.** Supplemental figure on distribution of restenosis rates (% decrease in lumen diameter per month) at the cephalic arch after the 2nd index to 3rd visits among patients treated with stent

**Additional file 8.** Supplemental figure on distribution of restenosis rates (% decrease in lumen diameter per month) at the cephalic arch after the 3rd index to 4th visits among patients treated with stent

**Additional file 9.** Supplemental figure on distribution of restenosis rates (% decrease in lumen diameter per month) at the cephalic arch after the 2nd index to 3rd visits among patients treated with bare metal stent

**Additional file 10.** Supplemental figure on distribution of restenosis rates (% decrease in lumen diameter per month) at the cephalic arch after the 3rd index to 4th visits among patients treated with bare metal stent

**Additional file 11.** Supplemental figure on distribution of restenosis rates (% decrease in lumen diameter per month) at the cephalic arch after the 2nd index to 3rd visits among patients treated with stent graft

**Additional file 12.** Supplemental figure on distribution of restenosis rates (% decrease in lumen diameter per month) at the cephalic arch after the 2nd index to 3rd visits among patients treated with stent at the 1st index visit and angioplasty at the 2nd index visit

**Additional file 13.** Supplemental figure on distribution of restenosis rates (% decrease in lumen diameter per month) at the cephalic arch after the 3rd index to 4th visits among patients treated with stent at the 1st index visit and angioplasty at the 3rd index visit

**Additional file 14.** Supplemental figure on distribution of restenosis rates (% decrease in lumen diameter per month) at the cephalic arch after the 2nd index to 3rd visits among patients treated with stent at the 1st index visit and standard angioplasty at the 2nd index visit

**Additional file 15.** Supplemental figure on distribution of restenosis rates (% decrease in lumen diameter per month) at the cephalic arch after the 3rd index to 4th visits among patients treated with stent at the 1st index visit and standard angioplasty at the 3rd index visit

**Additional file 16.** Supplemental figure on distribution of restenosis rates (% decrease in lumen diameter per month) at the cephalic arch after the 2nd index to 3rd visits among patients treated with stent at the 1st index visit and high-pressure angioplasty at the 2nd index visit

**Additional file 17.** Supplemental figure on distribution of restenosis rates (% decrease in lumen diameter per month) at the cephalic arch after the 3rd index to 4th visits among patients treated with stent at the 1st index visit and high-pressure angioplasty at the 3rd index visit

**Additional file 18.** Supplemental figure on distribution of restenosis rates (% decrease in lumen diameter per month) at the cephalic arch after the 2nd index to 3rd visits among patients treated with bare metal stent at the 1st index visit and angioplasty at the 2nd index visit

**Additional file 19.** Supplemental figure on distribution of restenosis rates (% decrease in lumen diameter per month) at the cephalic arch after the 3rd index to 4th visits among patients treated with bare metal stent at the 1st index visit and angioplasty at the 3rd index visit

**Additional file 20.** Supplemental figure on distribution of restenosis rates (% decrease in lumen diameter per month) at the cephalic arch after the 2nd index to 3rd visits among patients treated with stent graft at the 1st index visit and angioplasty at the 2nd index visit

**Additional file 21.** Supplemental figure on distribution of restenosis rates (% decrease in lumen diameter per month) at the cephalic arch after the 3rd index to 4th visits among patients treated with stent graft at the 1st index visit and angioplasty at the 3rd index visit

## Data Availability

The dataset used for this analysis is not publicly available. The data utilized was obtained from a private electronic medical record system, which is restricted to use by only authorized employees. Reasonable requests to access the study dataset might be considered under executed contractual agreements between Fresenius Medical Care and an external individual’s institution. Requests to access the dataset can be sent to the author J.W.L.

## Abbreviations

ATM: Atmospheres
AVF: Arteriovenous fistula
AVG: Arteriovenous graft
BCAVF: Brachial artery to cephalic vein arteriovenous fistula
IRB: Independent Review Board
KRT: Kidney replacement therapy
