# Supplementary figures and images for: Cephalic arch restenosis rates in hemodialysis patients with brachiocephalic fistulae: a retrospective multicenter analysis of 3301 patients
Source: BMC Nephrol. 2022 Mar 18;23:109. doi: 10.1186/s12882-022-02728-4 (PMC8932324; doi:10.1186/s12882-022-02728-4)

### Angioplasty only, 2nd (index) to 3rd visit

N = 1513

Min = 0

Max = 1677.5

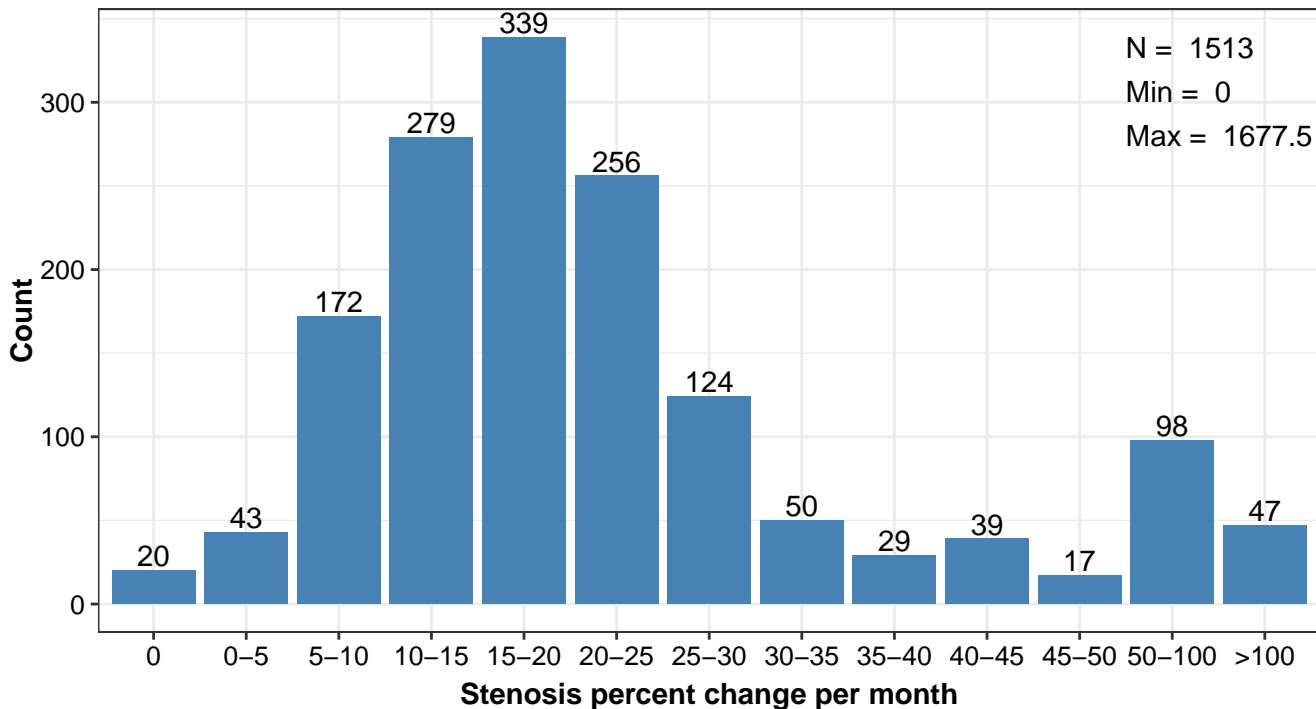

Supplement: Supplementary file 1 — Additional file 1. Supplemental figure on distribution of restenosis rates (% decrease in lumen diameter per month) at the cephalic arch after the 2nd index to 3rd visits among patients treated with angioplasty [file 12882_2022_2728_MOESM1_ESM.pdf]

# Angioplasty only, 3rd (index) to 4th visit

N = 901

Min = 0

Max = 1143.8

Count

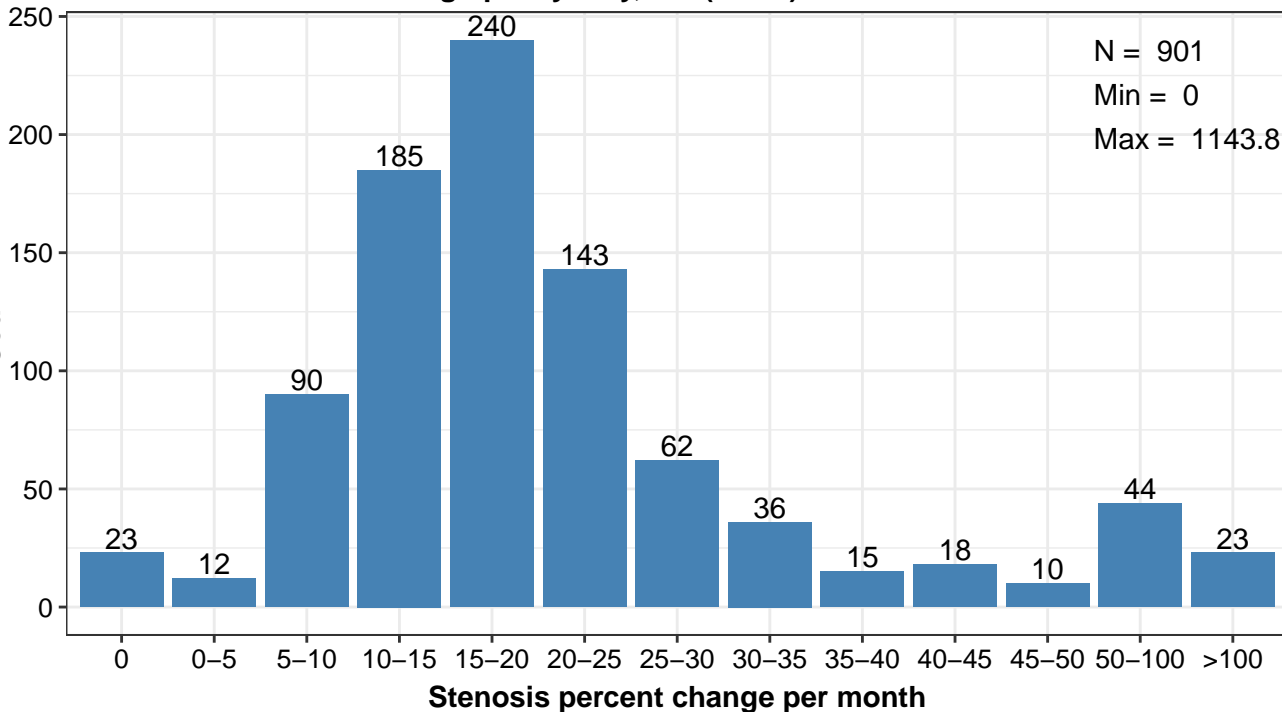

Supplement: Supplementary file 2 — Additional file 2. Supplemental figure on distribution of restenosis rates (% decrease in lumen diameter per month) at the cephalic arch after the 3rd index to 4th visits among patients treated with angioplasty [file 12882_2022_2728_MOESM2_ESM.pdf]

# Standard angioplasty, 2nd (index) to 3rd visit

N = 1172

Min = 0

Max = 1677.5

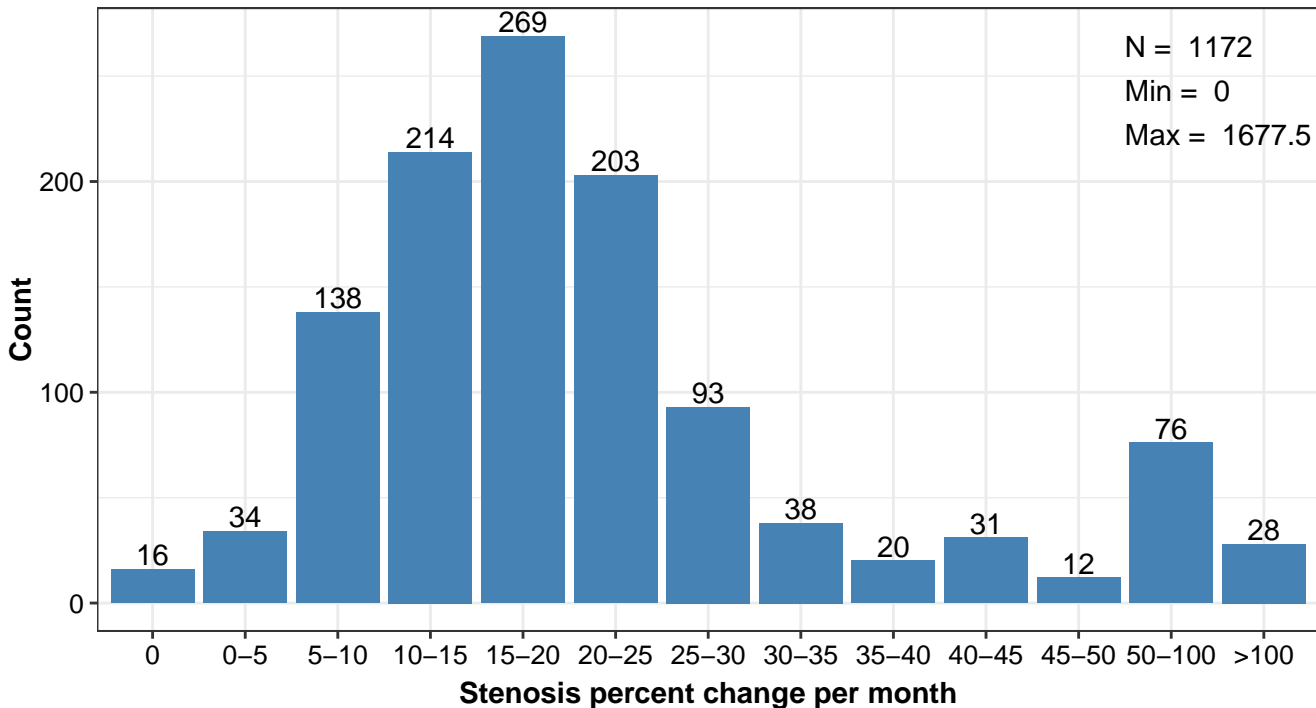

Supplement: Supplementary file 3 — Additional file 3. Supplemental figure on distribution of restenosis rates (% decrease in lumen diameter per month) at the cephalic arch after the 2nd index to 3rd visits among patients treated with standard angioplasty [file 12882_2022_2728_MOESM3_ESM.pdf]

### Standard angioplasty, 3rd (index) to 4th visit

N = 687

Min = 0

Max = 508.3

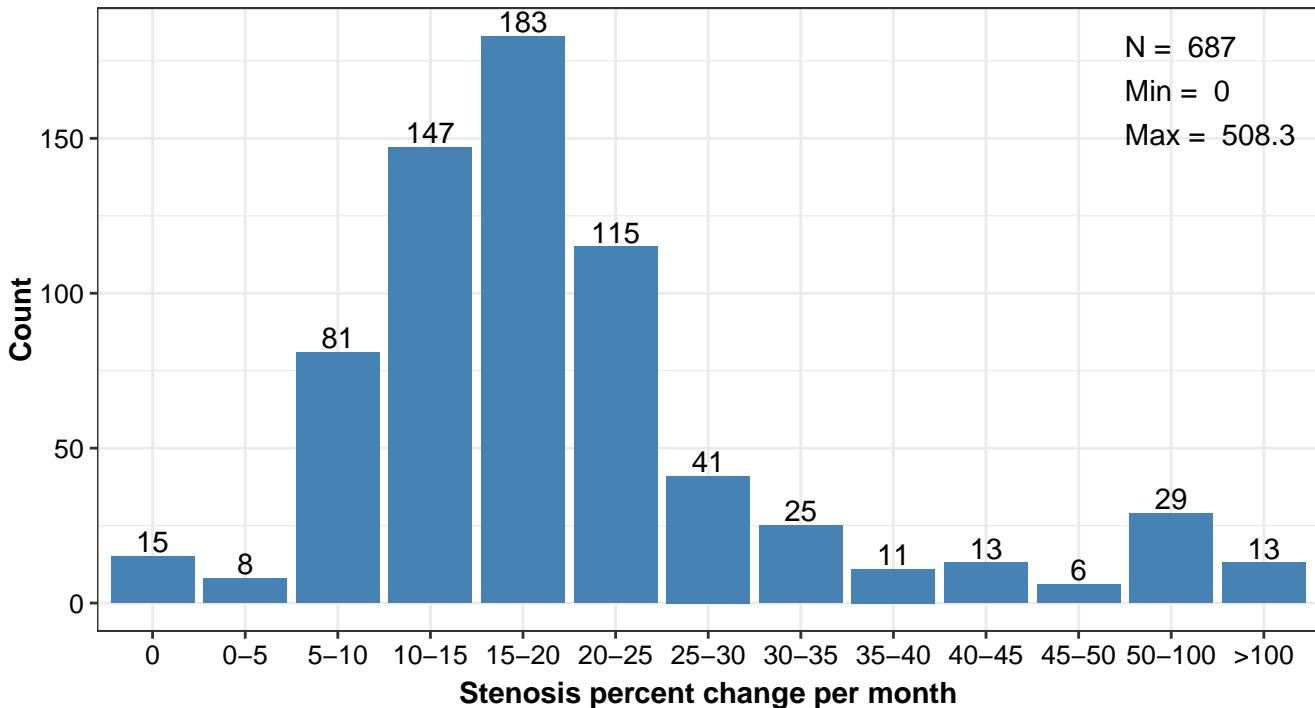

Supplement: Supplementary file 4 — Additional file 4. Supplemental figure on distribution of restenosis rates (% decrease in lumen diameter per month) at the cephalic arch after the 3rd index to 4th visits among patients treated with standard angioplasty [file 12882_2022_2728_MOESM4_ESM.pdf]

# High-pressure angioplasty, 2nd (index) to 3rd visit

N = 341

Min = 0

Max = 991.2

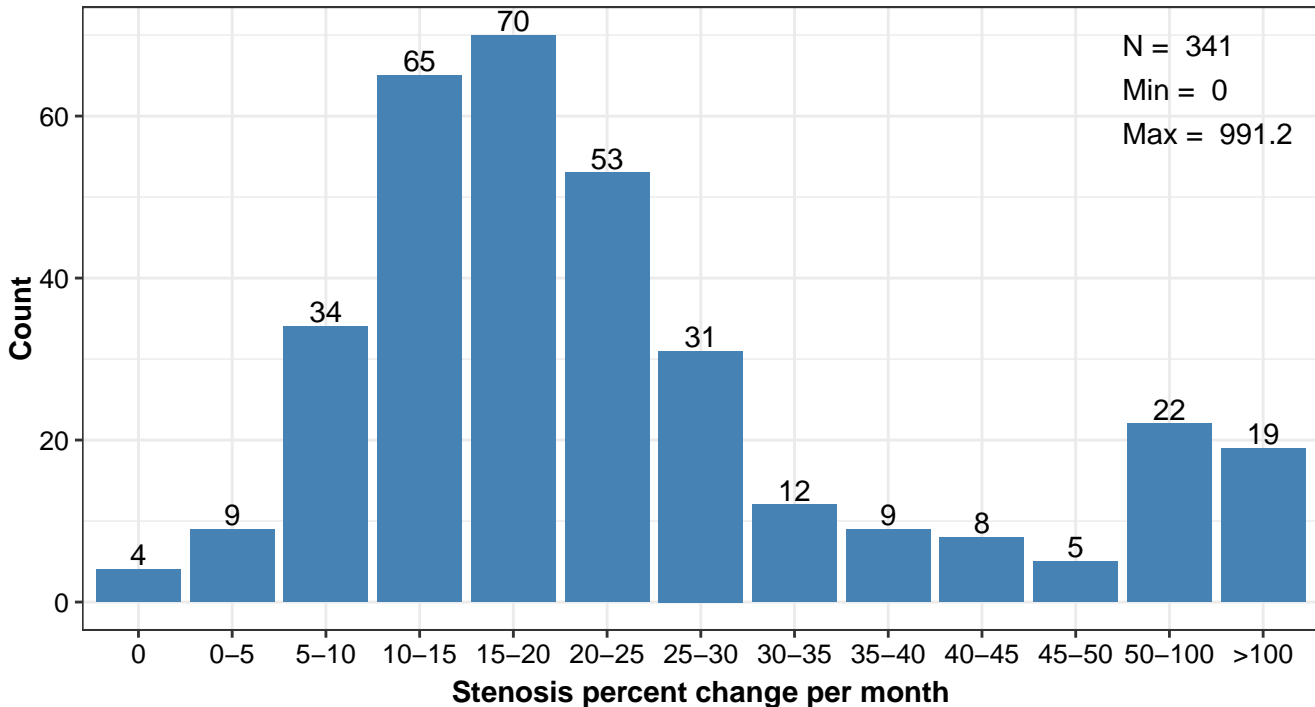

Supplement: Supplementary file 5 — Additional file 5. Supplemental figure on distribution of restenosis rates (% decrease in lumen diameter per month) at the cephalic arch after the 2nd index to 3rd visits among patients treated with high-pressure angioplasty [file 12882_2022_2728_MOESM5_ESM.pdf]

# High-pressure angioplasty, 3rd (index) to 4th visit

N = 214

Min = 0

Max = 1143.8

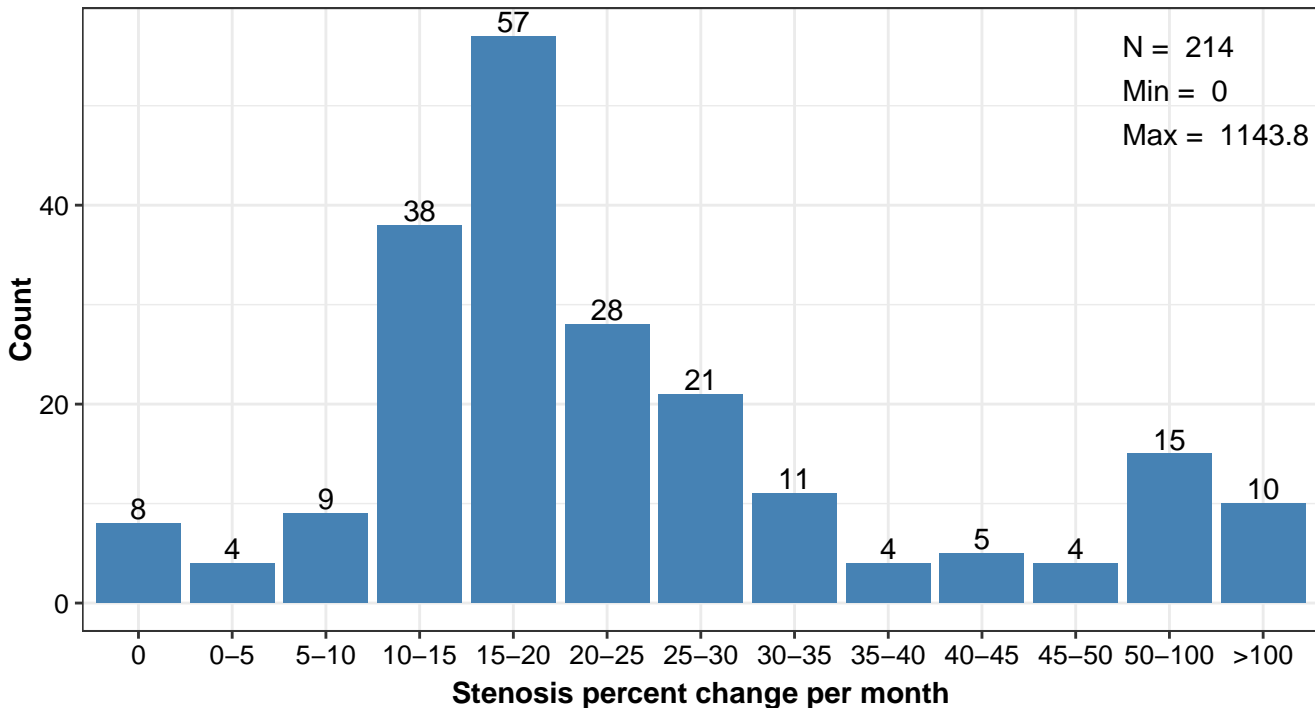

Supplement: Supplementary file 6 — Additional file 6. Supplemental figure on distribution of restenosis rates (% decrease in lumen diameter per month) at the cephalic arch after the 3rd index to 4th visits among patients treated with high-pressure angioplasty [file 12882_2022_2728_MOESM6_ESM.pdf]

**Stent, 2nd (index) to 3rd visit**

N = 48  
Min = 0  
Max = 283.2

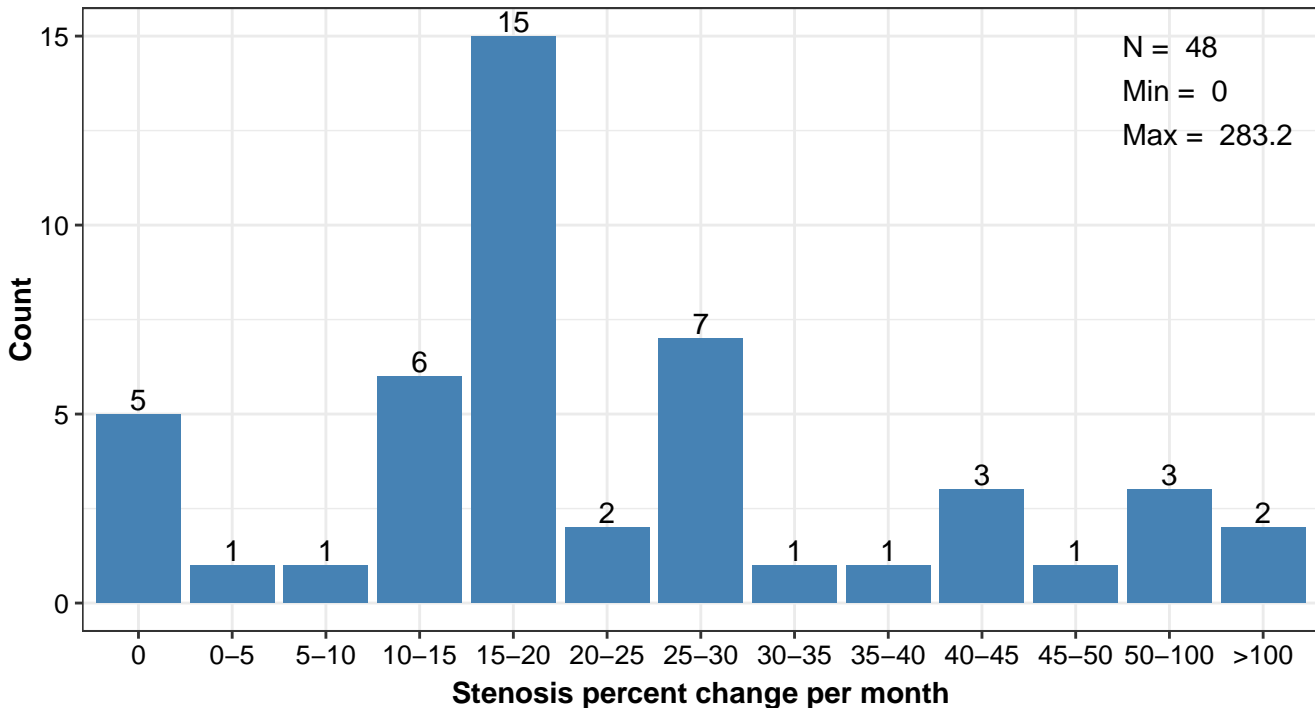

Supplement: Supplementary file 7 — Additional file 7. Supplemental figure on distribution of restenosis rates (% decrease in lumen diameter per month) at the cephalic arch after the 2nd index to 3rd visits among patients treated with stent [file 12882_2022_2728_MOESM7_ESM.pdf]

**Stent, 3rd (index) to 4th visit**

N = 5

Min = 12.8

Max = 28.5

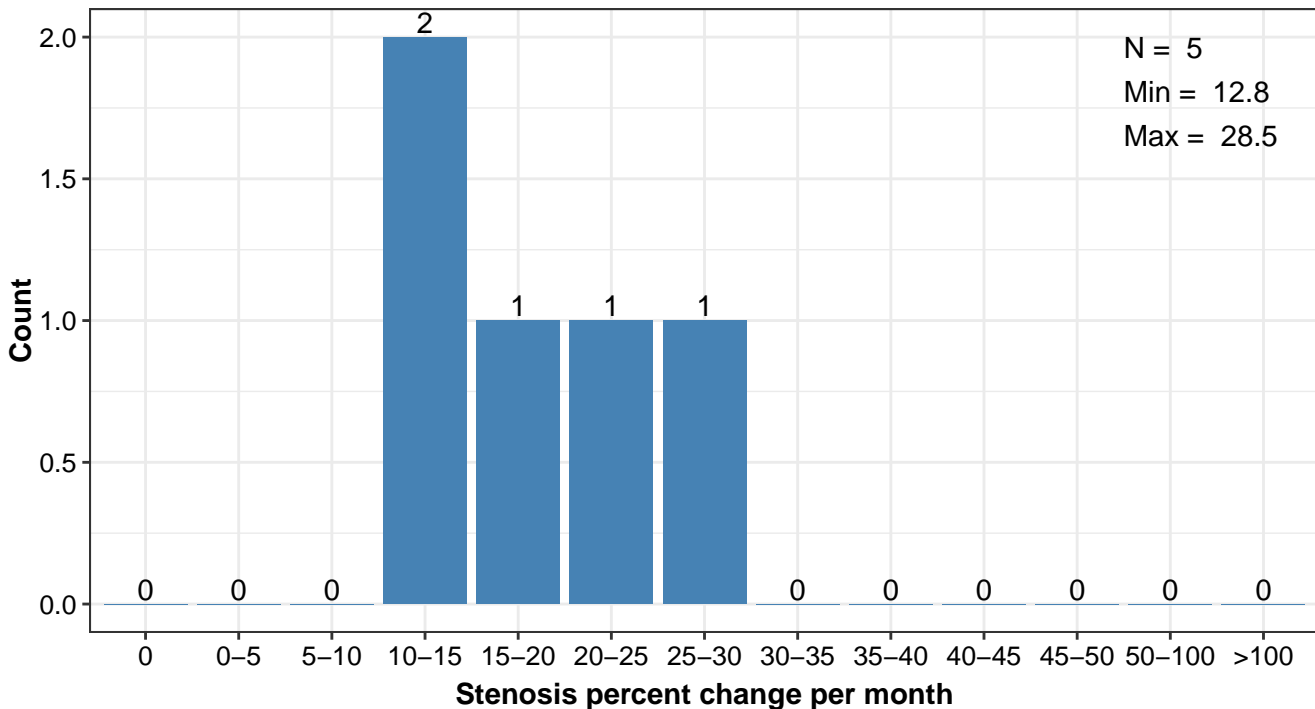

Supplement: Supplementary file 8 — Additional file 8. Supplemental figure on distribution of restenosis rates (% decrease in lumen diameter per month) at the cephalic arch after the 3rd index to 4th visits among patients treated with stent [file 12882_2022_2728_MOESM8_ESM.pdf]

# Bare metal stent, 2nd (index) to 3rd visit

N = 39

Min = 0

Max = 283.2

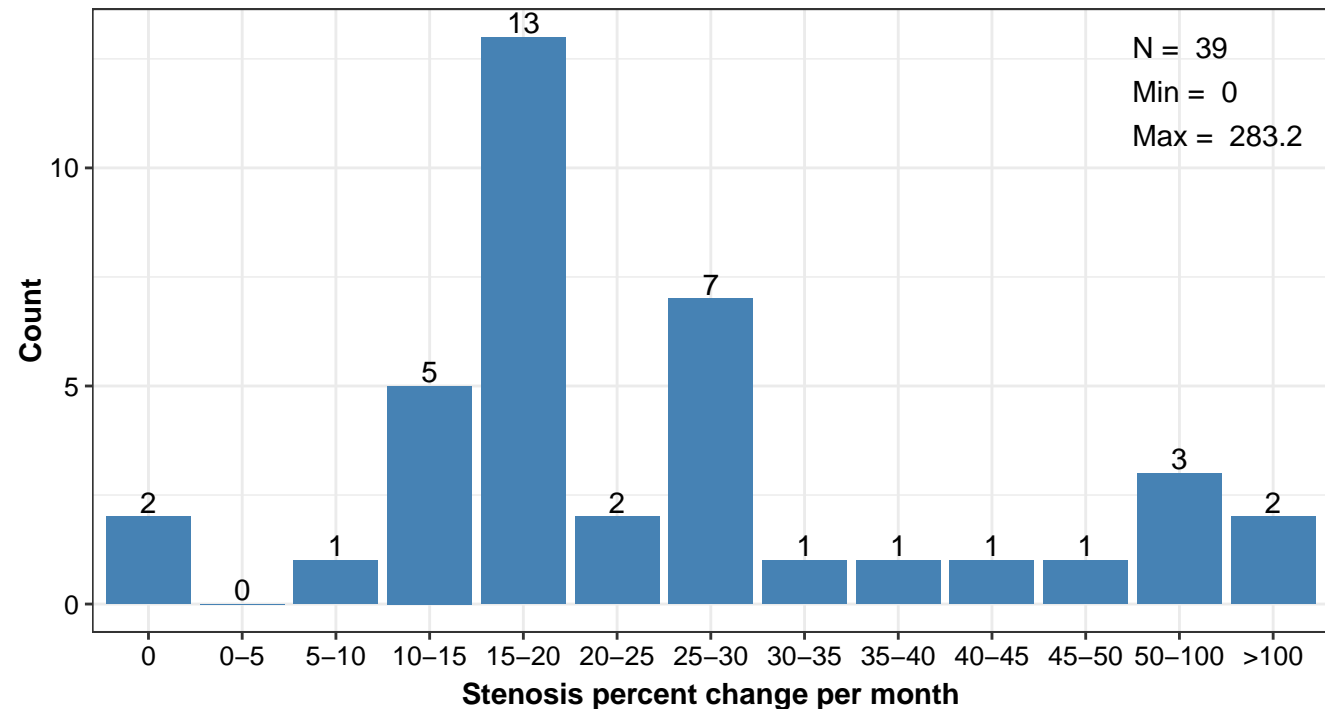

Supplement: Supplementary file 9 — Additional file 9. Supplemental figure on distribution of restenosis rates (% decrease in lumen diameter per month) at the cephalic arch after the 2nd index to 3rd visits among patients treated with bare metal stent [file 12882_2022_2728_MOESM9_ESM.pdf]

**Bare metal stent, 3rd (index) to 4th visit**

N = 5

Min = 12.8

Max = 28.5

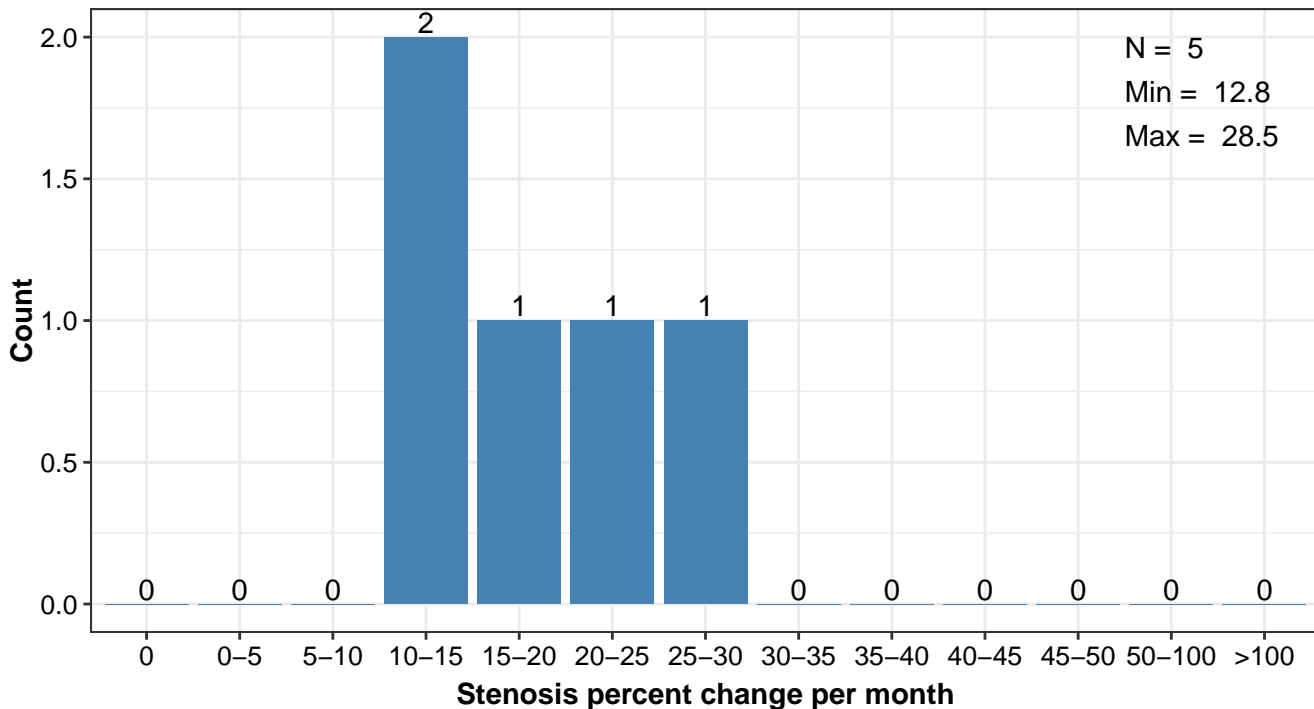

Supplement: Supplementary file 10 — Additional file 10. Supplemental figure on distribution of restenosis rates (% decrease in lumen diameter per month) at the cephalic arch after the 3rd index to 4th visits among patients treated with bare metal stent [file 12882_2022_2728_MOESM10_ESM.pdf]

**Stent graft, 2nd (index) to 3rd visit**

N = 9  
Min = 0  
Max = 44.7

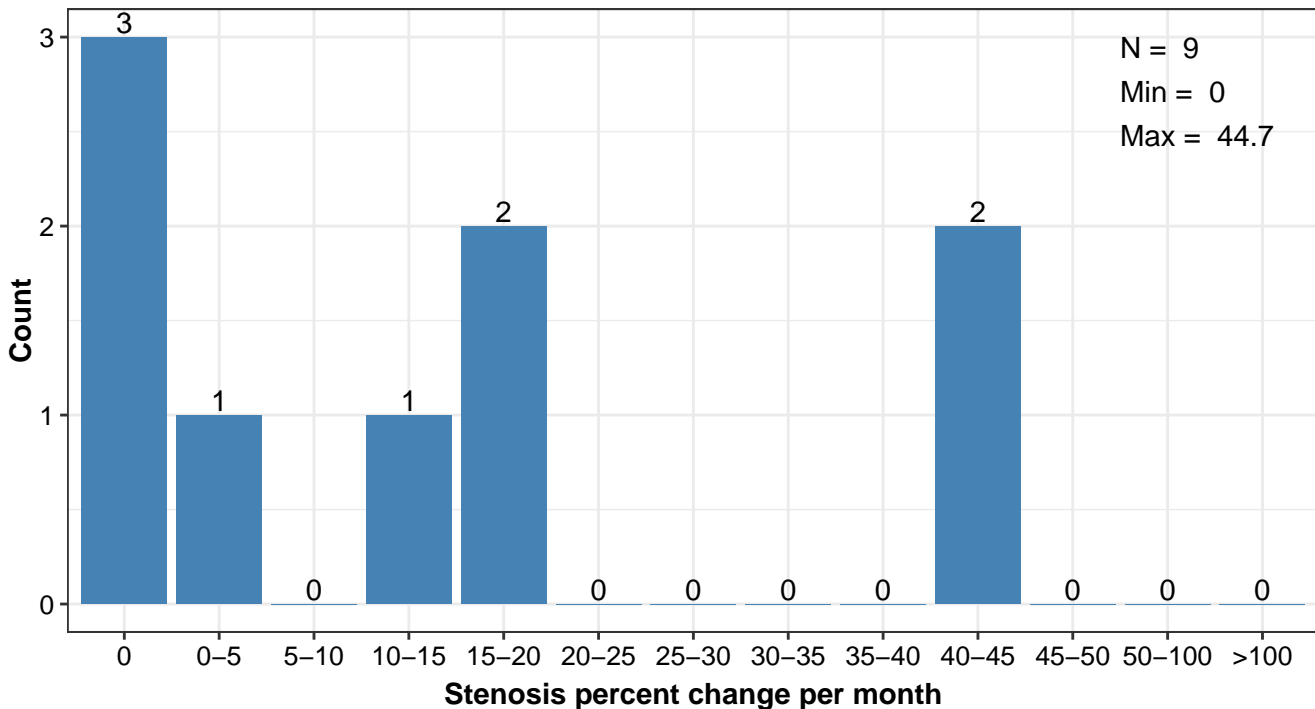

Supplement: Supplementary file 11 — Additional file 11. Supplemental figure on distribution of restenosis rates (% decrease in lumen diameter per month) at the cephalic arch after the 2nd index to 3rd visits among patients treated with stent graft [file 12882_2022_2728_MOESM11_ESM.pdf]

# Stent 1st index visit, and angioplasty 2nd (index) to 3rd visit

N = 410  
Min = 0  
Max = 2135

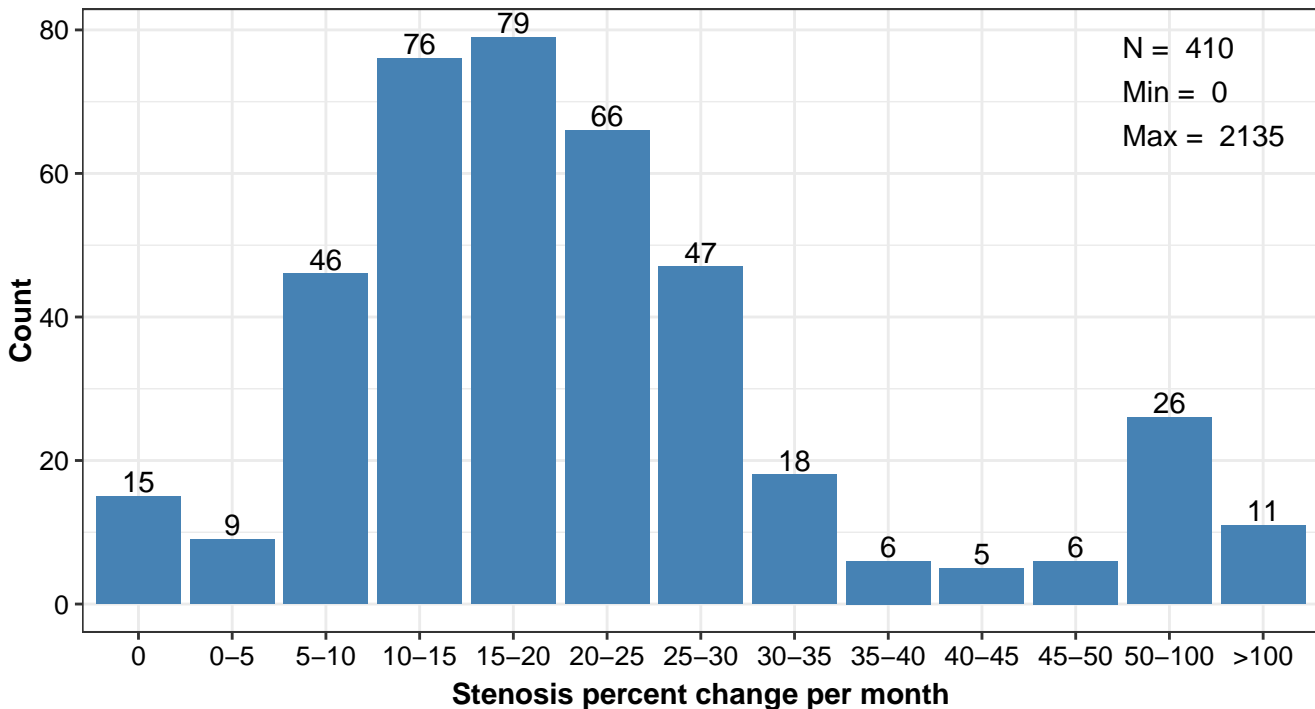

Supplement: Supplementary file 12 — Additional file 12. Supplemental figure on distribution of restenosis rates (% decrease in lumen diameter per month) at the cephalic arch after the 2nd index to 3rd visits among patients treated with stent at the 1st index visit and angioplasty at the 2nd index visit [file 12882_2022_2728_MOESM12_ESM.pdf]

# Stent 1st index visit, and angioplasty 3rd (index) to 4th visit

N = 191

Min = 0

Max = 366

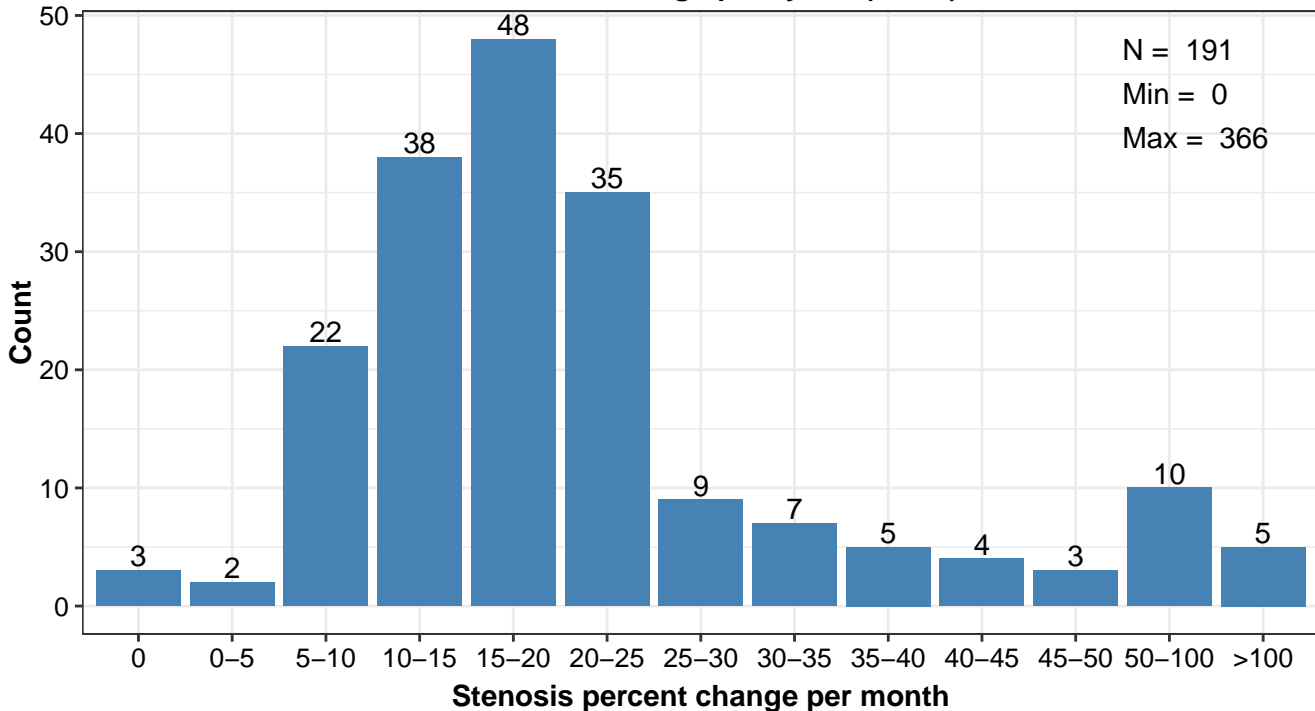

Supplement: Supplementary file 13 — Additional file 13. Supplemental figure on distribution of restenosis rates (% decrease in lumen diameter per month) at the cephalic arch after the 3rd index to 4th visits among patients treated with stent at the 1st index visit and angioplasty at the 3rd index visit [file 12882_2022_2728_MOESM13_ESM.pdf]

# Stent 1st index visit, and standard angioplasty 2nd (index) to 3rd visit

N = 337  
Min = 0  
Max = 2135

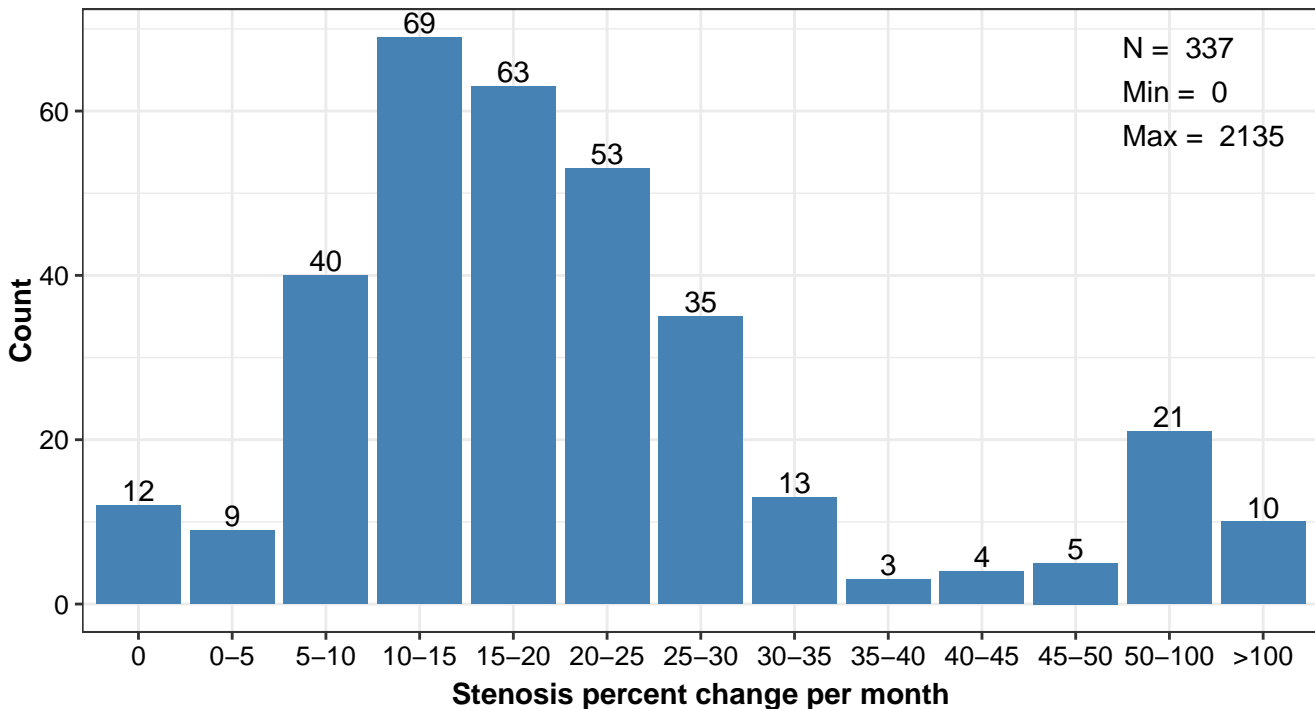

Supplement: Supplementary file 14 — Additional file 14. Supplemental figure on distribution of restenosis rates (% decrease in lumen diameter per month) at the cephalic arch after the 2nd index to 3rd visits among patients treated with stent at the 1st index visit and standard angioplasty at the 2nd index visit [file 12882_2022_2728_MOESM14_ESM.pdf]

# Stent 1st index visit, and standard angioplasty 3rd (index) to 4th visit

N = 155

Min = 0

Max = 152.5

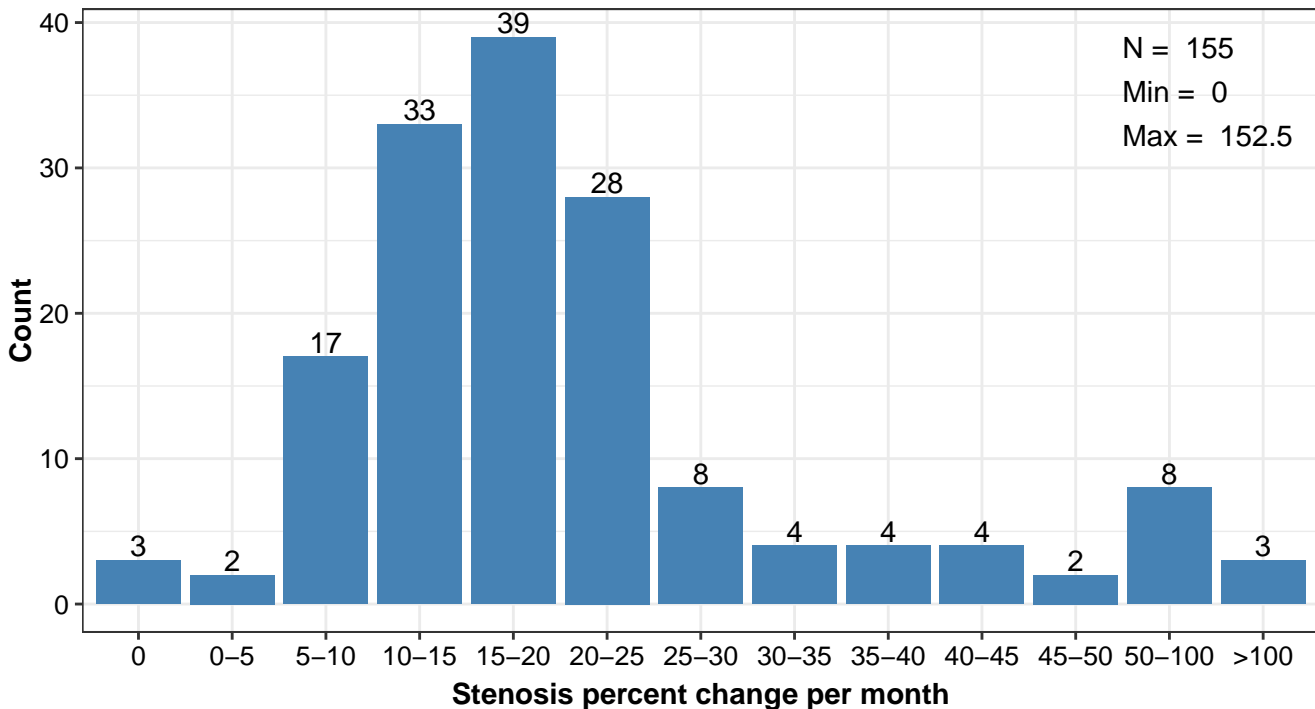

Supplement: Supplementary file 15 — Additional file 15. Supplemental figure on distribution of restenosis rates (% decrease in lumen diameter per month) at the cephalic arch after the 3rd index to 4th visits among patients treated with stent at the 1st index visit and standard angioplasty at the 3rd index visit [file 12882_2022_2728_MOESM15_ESM.pdf]

# Stent 1st index visit, and high-pressure angioplasty 2nd (index) to 3rd visit

N = 73

Min = 0

Max = 261.4

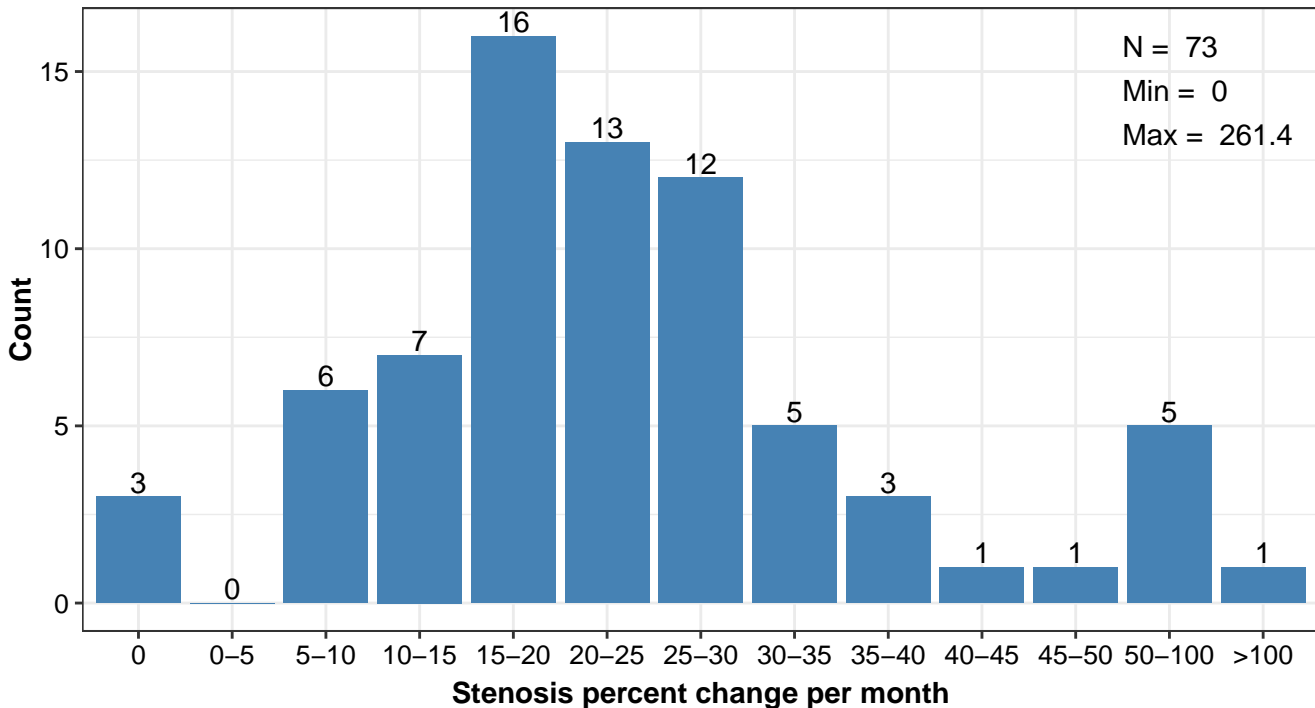

Supplement: Supplementary file 16 — Additional file 16. Supplemental figure on distribution of restenosis rates (% decrease in lumen diameter per month) at the cephalic arch after the 2nd index to 3rd visits among patients treated with stent at the 1st index visit and high-pressure angioplasty at the 2nd index visit [file 12882_2022_2728_MOESM16_ESM.pdf]

# Stent 1st index visit, and high-pressure angioplasty 3rd (index) to 4th visit

N = 36  
Min = 6.1  
Max = 366

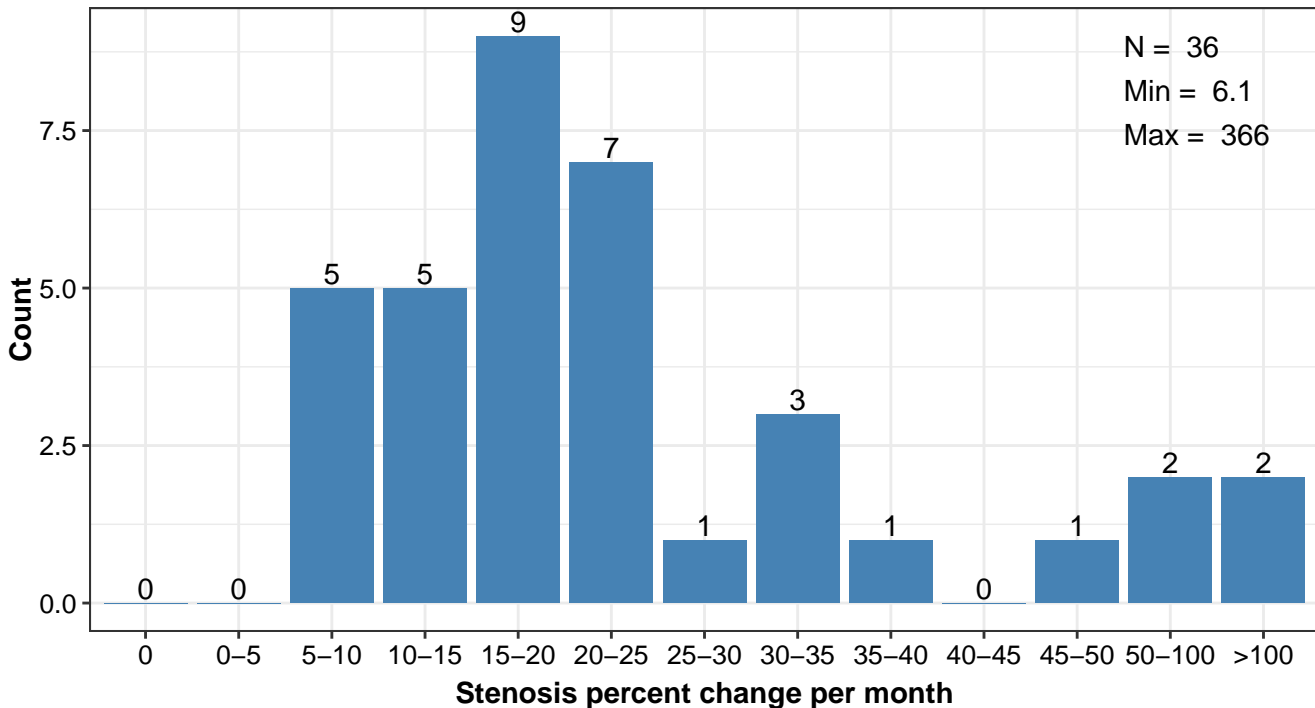

Supplement: Supplementary file 17 — Additional file 17. Supplemental figure on distribution of restenosis rates (% decrease in lumen diameter per month) at the cephalic arch after the 3rd index to 4th visits among patients treated with stent at the 1st index visit and high-pressure angioplasty at the 3rd index visit [file 12882_2022_2728_MOESM17_ESM.pdf]

# Bare metal stent 1st index visit, and angioplasty 2nd (index) to 3rd visit

N = 332

Min = 0

Max = 2135

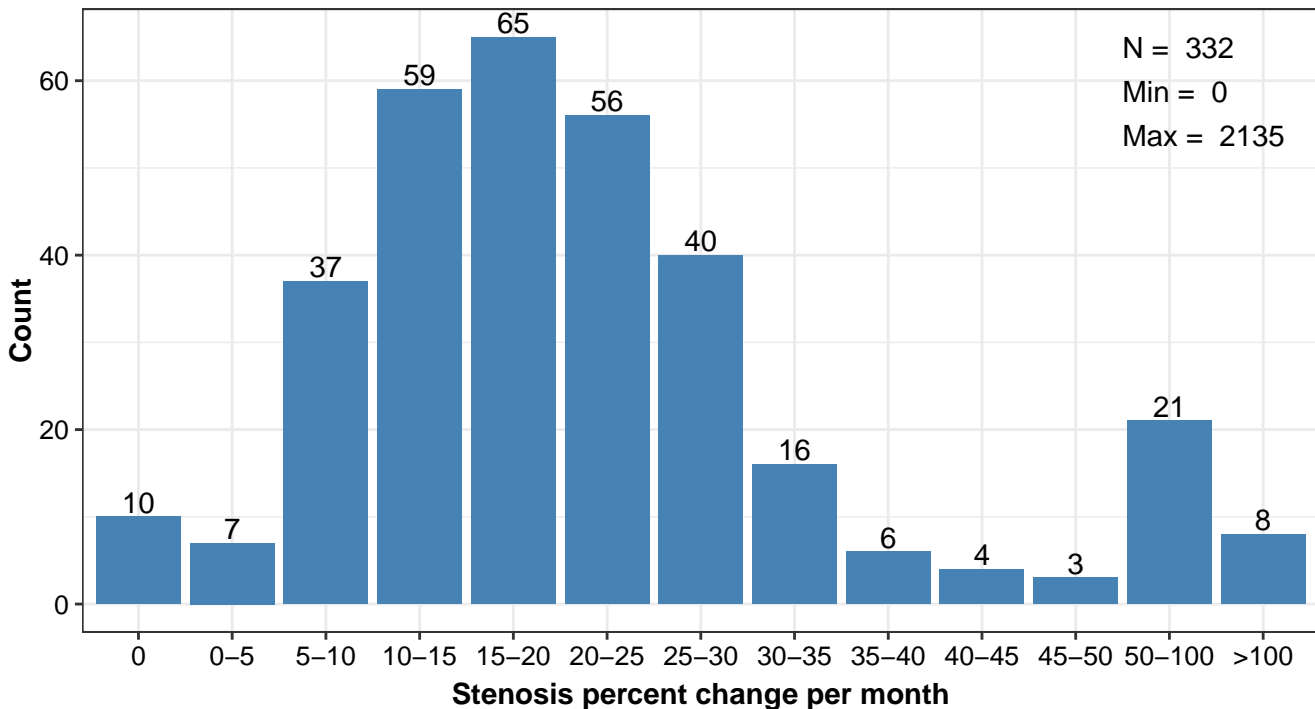

Supplement: Supplementary file 18 — Additional file 18. Supplemental figure on distribution of restenosis rates (% decrease in lumen diameter per month) at the cephalic arch after the 2nd index to 3rd visits among patients treated with bare metal stent at the 1st index visit and angioplasty at the 2nd index visit [file 12882_2022_2728_MOESM18_ESM.pdf]

# Bare metal stent 1st index visit, and angioplasty 3rd (index) to 4th visit

N = 149

Min = 0

Max = 366

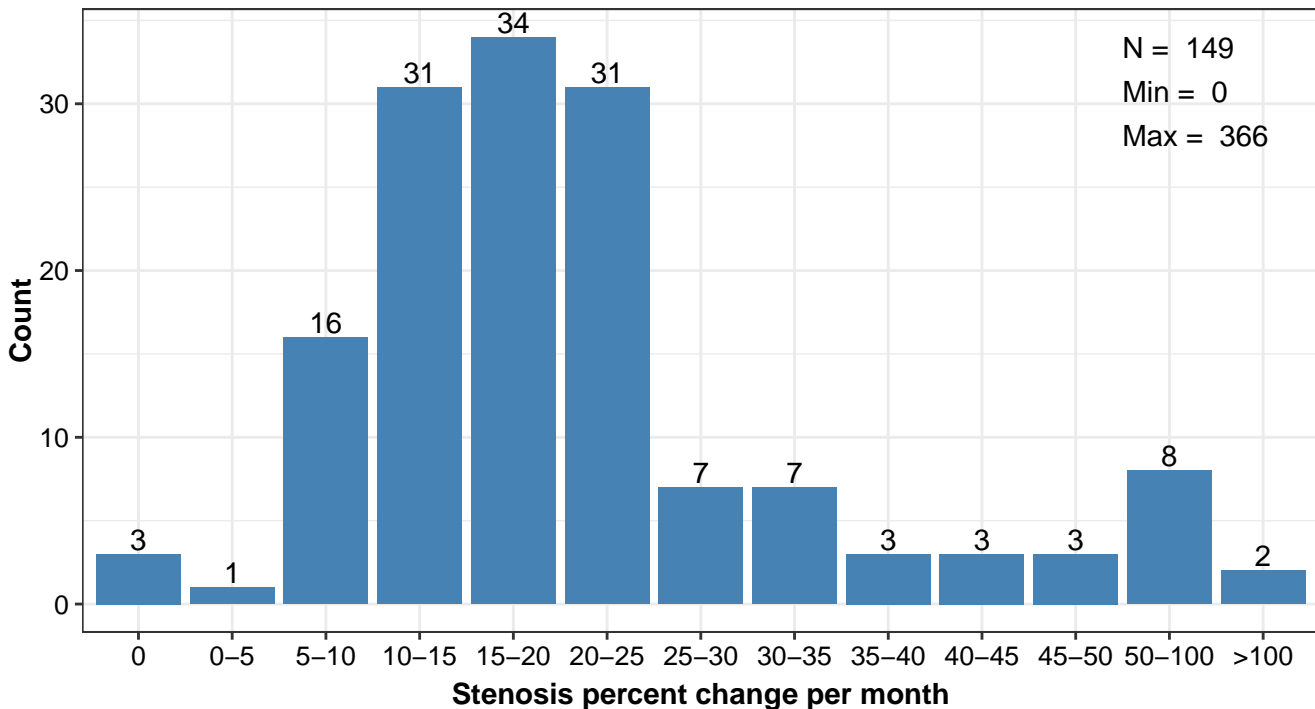

Supplement: Supplementary file 19 — Additional file 19. Supplemental figure on distribution of restenosis rates (% decrease in lumen diameter per month) at the cephalic arch after the 3rd index to 4th visits among patients treated with bare metal stent at the 1st index visit and angioplasty at the 3rd index visit [file 12882_2022_2728_MOESM19_ESM.pdf]

# Stent graft 1st index visit, and angioplasty 2nd (index) to 3rd visit

N = 76  
Min = 0  
Max = 228.8

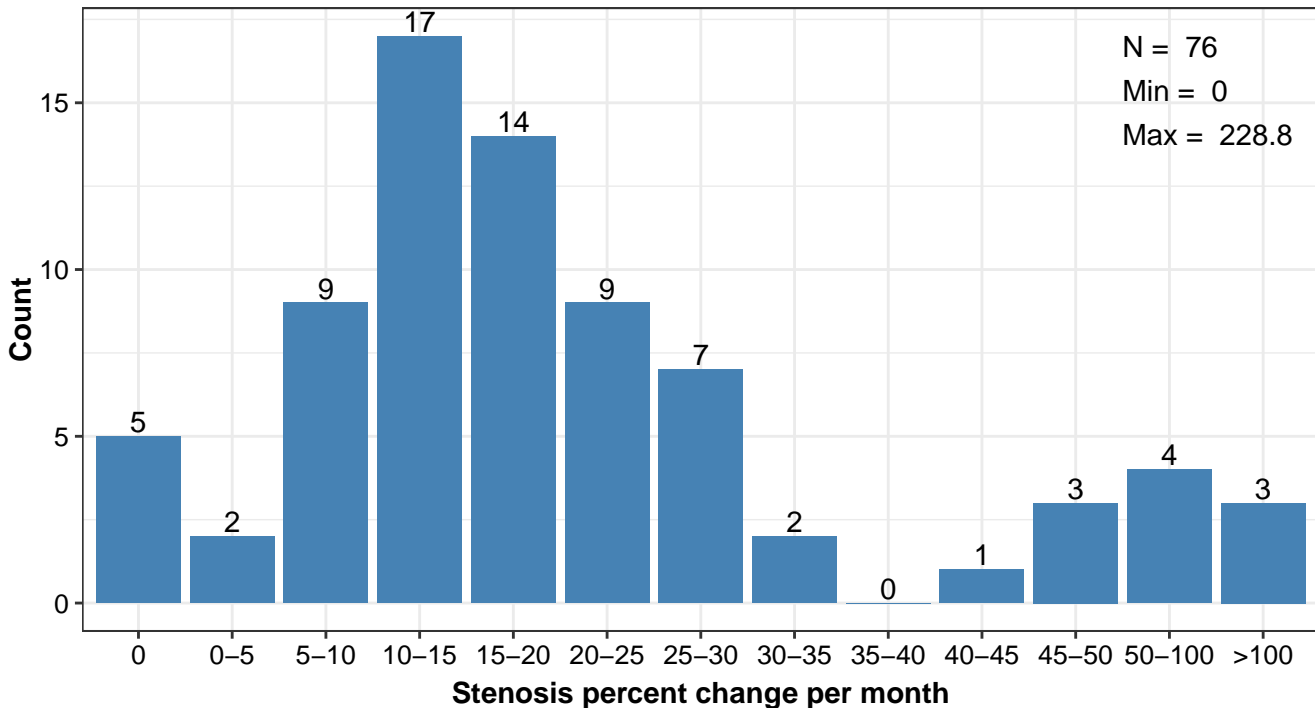

Supplement: Supplementary file 20 — Additional file 20. Supplemental figure on distribution of restenosis rates (% decrease in lumen diameter per month) at the cephalic arch after the 2nd index to 3rd visits among patients treated with stent graft at the 1st index visit and angioplasty at the 2nd index visit [file 12882_2022_2728_MOESM20_ESM.pdf]

# Stent graft 1st index visit, and angioplasty 3rd (index) to 4th visit

N = 42

Min = 5

Max = 152.5

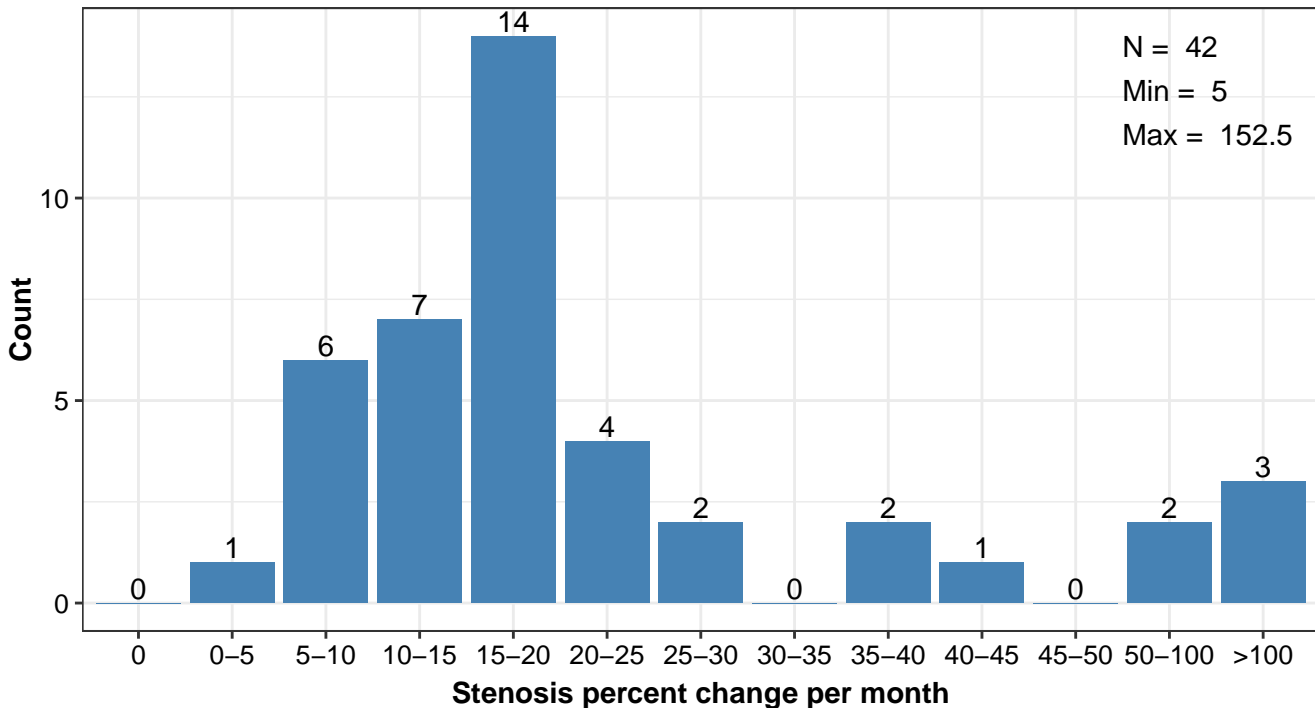

Supplement: Supplementary file 21 — Additional file 21. Supplemental figure on distribution of restenosis rates (% decrease in lumen diameter per month) at the cephalic arch after the 3rd index to 4th visits among patients treated with stent graft at the 1st index visit and angioplasty at the 3rd index visit [file 12882_2022_2728_MOESM21_ESM.pdf]
